# Childhood psychopathology mediates associations between childhood adversities and multiple health risk behaviours in adolescence: analysis using the ALSPAC birth cohort

**DOI:** 10.1111/jcpp.13379

**Published:** 2021-02-22

**Authors:** David Troy, Abigail Russell, Judi Kidger, Caroline Wright

**Affiliations:** ^1^ School of Population Health Sciences Bristol Medical School University of Bristol Bristol UK; ^2^ Children and Young People's Mental Health Research Collaboration College of Medicine and Health University of Exeter Exeter UK

**Keywords:** Adverse childhood experiences, multiple health risk behaviours, adolescence, psychosocial attributes, mediation analysis, structural equation modelling, ALSPAC, UK birth cohort study

## Abstract

**Background:**

Childhood adversity strongly predicts adolescent multiple health risk behaviours (MRBs) such as alcohol/tobacco use, self‐harm and physical inactivity, and both adversities and MRBs are associated with premature mortality and several chronic health conditions that are among the leading causes of death in adults. It is therefore important to understand the relationship between adversities and MRBs and what could mediate any association. The aim of this study was to explore whether childhood psychopathology mediates associations between adversities and MRBs.

**Methods:**

Participants were young people in the Avon Longitudinal Study of Parents and Children (ALSPAC) (*N* = 5,799). Using structural equation modelling, we explored the associations between adversities before 9 years and MRBs at age 16 years. We also explored potential mediating pathways through dimensional psychopathology measured by the Strength and Difficulties Questionnaire subscales at age 12 years.

**Results:**

There were strong positive associations between adversities and MRBs (β .25, 95% CI 0.20, 0.31, *p* < .001) suggesting that each additional adversity is associated with a 0.25 increase in number of MRBs out of 13 total risk behaviours. We found robust evidence of mediating pathways from adversities through conduct problems (β .05, 95% CI 0.03, 0.06, *p* < .001), hyperactivity/inattention (β .02, 95% CI 0.01, 0.03, *p* < .001) and peer relationship problems (β −.02, 95% CI −0.03, −0.02, *p* < .001) to MRBs.

**Conclusions:**

Increased conduct problems and hyperactivity/inattention appear to partially explain the relationship between adversities and MRBs. Peer relationship problems also appear to reduce the association between adversities and MRBs, and further research is needed to understand how to encourage peer connectivity without increasing risk of MRBs. These results suggest that interventions aimed at reducing MRBs among those exposed to childhood adversities could focus on prevention of behavioural problems.

## Introduction

The significance of early‐life experiences to health behaviours in adolescence and beyond is receiving increased attention. Childhood adversities (commonly called adverse childhood experiences or ACEs) are a leading cause of inequity in health (Sethi et al., 2013) and have high societal costs (Caspi et al., 2016) that are considered largely avoidable (Sethi et al., 2013). Childhood adversities are characterised by harms that affect children directly (e.g. physical, sexual abuse) and indirectly through their environment (e.g. parental substance use). Research has focused on the long‐term effects of single types of adversity (e.g. Bryant & Range, 1995; McCauley et al., 1997), but also on the cumulative impacts of multiple types of adversity, with studies showing a gradient of increasingly poor health associated with exposure to more types of adversity (Bellis et al., 2019; Hughes et al., 2017).

Childhood adversities are strongly associated with multiple health risk behaviours (MRBs) (Bellis, Hughes, Leckenby, Jones, et al., 2014; Bellis, Hughes, Leckenby, Perkins, & Lowey, 2014; Bellis, Lowey, Leckenby, Hughes, & Harrison, 2013; Hughes et al., 2017). Individuals exposed to higher numbers of adversities have an increased risk of engaging in MRBs in adolescence and adulthood including smoking (Anda et al., 1999; Ford et al., 2011), earlier age of drinking onset (Rothman, Edwards, Heeren, & Hingson, 2008), heavy alcohol consumption (Anda et al., 2002), substance use, high‐risk sexual behaviour, suicidal ideation and suicide (Felitti et al., 2019), physical inactivity (Kestilä et al., 2015) and sedentary behaviour (e.g. excessive TV viewing) (Iguacel, Gasch‐Gallén, Ayala‐Marín, De Miguel‐Etayo, & Moreno, 2020). There is evidence that MRBs are strongly associated with early‐life socioeconomic status (Kipping, Smith, Heron, Hickman, & Campbell, 2015); poorer educational attainment (Wright, Kipping, Hickman, Campbell, & Heron, 2018); and adverse health and social outcomes in early adulthood (Campbell et al., 2020). These MRBs were chosen owing to the strong evidence linking them to poor health. There is increasing evidence that childhood adversities are important determinants of adult morbidity and mortality with increased odds (odds ratio: 1.4) of coronary heart disease, stroke and diabetes among those with exposure to 4‐6 adversities compared to those with no exposure (Gilbert et al., 2015; Teicher & Samson, 2016). Childhood adversities and adolescent MRBs are both associated with several of the leading causes of death in adults including ischaemic heart disease and cancer (Felitti et al., 2019) as well as with premature mortality in the range of a 20‐year life expectancy reduction for individuals reporting 6 adversities compared to individuals reporting none (Brown et al., 2009; Kelly‐Irving et al., 2013).

Studies are increasingly demonstrating how childhood exposure to repeated or chronic stress can lead to changes in the development of the nervous system resulting in impaired emotional, social and cognitive functioning (Danese & McEwen, 2012; Pechtel & Pizzagalli, 2011). Childhood adversities have been shown to be associated with poorer psychological functioning (Brown et al., 2017; McKelvey, Edge, Mesman, Whiteside‐Mansell, & Bradley, 2018), and psychopathology increases MRB risk (Katon et al., 2010; Sentse, Kretschmer, De Haan, & Prinzie, 2017); as such, childhood psychopathology may lie on the causal pathway between childhood adversities and MRBs. Identifying intermediary agents on these causal pathways will provide targets for intervention that could interrupt the progression from childhood adversities to adolescent MRBs, with the potential to prevent a cascade of negative outcomes. Studies have investigated the mediating influence of individual domains of psychopathology between childhood adversities and individual or relatively few health risk behaviours (Kim et al., 2018; Wardell, Strang, & Hendershot, 2016). To our knowledge, this is the first study that tests a broad range of child psychopathology domains as a mediator of the relationship between adversities and a wide range of MRBs. In this study, we specifically explored associations between childhood adversity before 9 years and adolescent MRBs at 16 years and examined whether symptoms of childhood psychopathology at 12 years mediated this relationship.

## Methods

### Sample

Participants were drawn from the Avon Longitudinal Study of Parents and Children (ALSPAC), an ongoing prospective observational population‐based cohort. Pregnant women resident in Avon, UK with expected dates of delivery 01/04/1991 to 31/12/1992 were invited to take participate (Boyd et al., 2013; Fraser et al., 2013). Of 14,541 initial pregnancies, 13,988 children (singletons and twins) were alive at 1 year. The sample for the current study comprised young people who had complete data on the mediators (*N* = 5,799). Complete data on exposure, mediators and outcome variables were available for 1,348 participants (the complete case sample; see Figure S1 for a flow chart detailing sample definition). Please note that the study website contains details of all the data that is available through a fully searchable data dictionary and variable search tool: http://www.bristol.ac.uk/alspac/researchers/our‐data/ (ALSPAC, 2016).

### Ethical considerations

Ethical approval for the study was obtained from the ALSPAC Ethics and Law Committee and the Local Research Ethics Committees. Informed consent for the use of data collected via questionnaires and clinics was obtained from participants following the recommendations of the ALSPAC Ethics and Law Committee at the time.

### Measures

#### Exposure: Adversities

Mothers, partners and the study child were asked 288 questions over 27 data collection points about the child’s exposure to nine adversities up to 9 years old (this age cut‐off was used due to wanting clear delineation between the time our exposure occurred and when mediators were measured at age 12). Adversities were child sexual, physical or emotional abuse; parent substance use; parent mental health problems or suicide attempt; violence between parents; parental separation; parental criminal conviction; and child bullying. Table S1 contains definitions of adversities (together with the number of contributing questions), and these are very similar to the most commonly used adversity definitions (WHO, 2015). Adversities were derived as in previous papers (Houtepen, Heron, Suderman, Tilling, & Howe, 2018; Russell et al., 2019) (see Table S2 for more details). Most adversities were operationalised by the responses to these questions, for example a child experienced physical abuse if they were kicked, punched, hit, smacked by an adult in the family. Additionally, some validated measures were used as the cut‐off for parents having mental health problems, for example >12 on the Edinburgh Postnatal Depression Scale. Adversities were considered present if criteria were met at least once by the time the child was age 9 years which is an approach used elsewhere (WHO, 2015). As our exposure measure, we used a continuous score of the number of types of adversity experienced.

#### Outcome measure: MRBs

Measures of participation in thirteen distinct health risk behaviours defined as behaviours that are associated with poor health covering the domains of sexual health, criminal and antisocial behaviour, substance use, self‐harm, vehicle‐related injury risk and physical inactivity. A total MRB construct was created to get an overall picture of the risk profile of participants. Research suggests that health risk behaviours cluster together, indicating that these behaviours should not be viewed in isolation in this age group (Kwan, Arbour‐Nicitopoulos, Duku, & Faulkner, 2016). Health risk behaviours at the ages of 15 and 16 years were derived from participants’ responses at two timepoints as in previous papers (Kipping et al., 2015; Wright et al., 2018). The first was a self‐completed questionnaire issued during a clinic attended at age 15 years (median age 15 years and 5 months) and the second comprised responses to a postal questionnaire administered at age 16 years (median age 16 years and 7 months). Binary indicators were used for all behaviours and thresholds were chosen by a research team involved in a previous study (Kipping et al., 2015) due to the discrete nature of some behaviours (e.g. self‐harm) or the level of engagement likely to infer risk (e.g. alcohol consumption). For most of the behaviours, there are not defined thresholds of ‘risk’ by age group, physical activity was the exception to this. Additional information on how MRBs were derived and their thresholds is in Table S3. For the purposes of the analyses reported here, the total number of risk behaviours in the imputed sample score from 0 to 13 was derived for each participant (range 0–13, mean: 2.94 and standard error (*SE*): 0.04). Owing to the positive skew of the MRB score, we checked the residuals from a fully fitted model, and finding them to be normally distributed, did not make any transformations.

#### Mediators

The Strength and Difficulties Questionnaire (SDQ) parent report is a validated and reliable assessment of dimensional child psychopathology (Goodman, 2001). It is an instrument of 25 items in five subscales: conduct problems, hyperactivity/inattention, emotional symptoms, peer relationship problems and prosocial behaviours (questions asked in each subscale are in Table S4). Higher scores on the four subscales that report on negative behaviours reflect more significant problems, whereas higher scores on the prosocial behaviour subscale denote better social behaviour. We used each of the five subscales as separate mediators each containing five items (scores ranged from 0 to 10).

#### Potential confounders

We adjusted for potential confounders (common causes of both exposures and outcomes) that were, where possible, collected prior to our exposure measure: sex, parent’s highest social class, mother’s highest educational level reported during pregnancy, household income and housing tenure during pregnancy (for confounder categories see Table 1). We additionally controlled for Intelligence Quotient score at age 8 years, age of mother at birth and SDQ scores at age 9.

**Table 1:** Descriptive statistics of study variables in imputed and complete case samples

Variable	Imputed data		Complete case sample	
	(*N* = 5,799)		(*N* = 1,348)	
	*n*	% or mean (*SE*)	*n*	Mean (*SD*) % (*SE*)
Adversity score	3,037	1.13 (0.02)		1.02 (1.20)
MRB score	2,086	2.94 (0.04)		2.86 (1.90)
SDQ – Hyperactivity		2.61 (0.03)		2.20 (1.91)
SDQ – Emotional		1.37 (0.02)		1.28 (1.60)
SDQ – Conduct		1.10 (0.02)		0.92 (1.16)
SDQ – Peer		1.00 (0.02)		0.97 (1.46)
SDQ – Prosocial		8.41 (0.02)		8.49 (1.54)
Child sex (% female)	2,944	50.77 (0.66)	807	59.87 (1.34)
Housing tenure (% not owned/mortgaged)	912	16.11 (0.72)	104	7.71 (1.04)
Maternal education (%)				
Degree	4,556	19.82 (0.59)	1,348	27.23 (1.21)
A‐level		28.38 (0.67)		31.53 (1.27)
GCSE		34.70 (0.71)		31.45 (1.27)
<GCSE		17.10 (0.56)		9.79 (0.01)
Equivalised household income (quintiles, %)				
Highest (1)	4,288	25.44 (0.67)	1,348	31.97 (1.27)
2		23.53 (0.65)		26.48 (1.20)
3		20.85 (0.62)		20.70 (1.10)
4		17.54 (0.58)		13.50 (0.93)
Lowest (5)		12.64 (0.51)		7.34 (0.71)
Parents’ highest social class (%)				
Professional	4,394	18.80 (0.59)	1,348	24.70 (1.18)
Managerial/technical		46.34 (0.75)		48.52 (1.36)
Skilled nonmanual		23.08 (0.64)		20.03 (1.09)
Skilled manual		11.79 (0.49)		6.75 (0.68)
IQ at 8 years	4,459	106.0 (0.24)	1,348	111.33 (15.11)
Maternal age at child birth (years)	5,570	29.17 (0.06)	1,348	30.03 (4.22)

GCSE, General Certificate of Secondary Education; IQ, Intelligence Quotient; SDQ, Strengths and Difficulties Questionnaire.

Multiple imputation by chained equations was used to account for missing data (Royston & White, 2011). Little’s chi‐square test showed strong evidence (*p* < .001) that both adversity and MRB data were missing completely at random (additional missingness information in Table S10). Our imputation sample had complete data on all SDQ subscales (*N* = 5,799); as we were interested in childhood psychopathology as a potential mediating mechanism in our longitudinal study design, it was important that SDQ scores were collected after the adversities and prior to MRBs, meaning that we did not want to impute scores based on prior or subsequent SDQ measures. Imputation of each of the study variables used a bespoke combination of auxiliary variables (that are not included in the analysis model but provide additional information about the missing values) including alternative socioeconomic variables and earlier and later measures of adversities and MRBs. A number of data sets with a varying number of imputations were generated using the *ice* command in Stata/MP v15.1 (Statacorp, 2017). Monte Carlo errors were used to compare the probability that imputation would be reproducible. We compared the imputation of 100, 250, 500 and 750 data sets using White et al.’s rules of thumb (White, Royston, & Wood, 2011) with 750 data sets being chosen as the most appropriate number of imputed data sets. The main results reported are based on the imputed data, with complete case results reported in the Supporting Information.

### Analysis

Tetrachoric correlations between adversities and between MRBs were reported. A mediation model was fitted to determine whether the association between childhood adversities and adolescent MRBs was mediated by SDQ subscale scores at 12 years. We used a structural equation modelling approach to partition the association between adversity score, SDQ subscales and MRB score into direct and indirect effects with robust standard errors using the sem and nlcom commands in Stata/MP v15.1 (Gunzler, Chen, Wu, & Zhang, 2013). Sensitivity analyses removing physical inactivity and excessive TV viewing and adding SDQ at age 9 were conducted. Bootstrapping was used to estimate bias‐corrected 95% confidence intervals in complete case data (1,000 replications).

## Results

### Descriptive results

**Figure 1:**
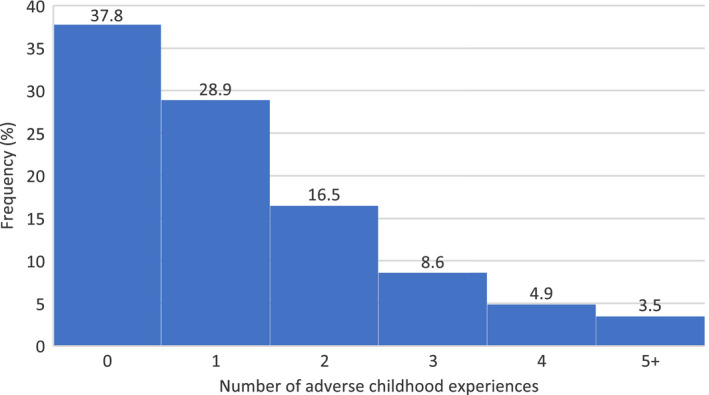
Number of adversities per child (imputed data *N* = 5,799). Notes: 750 imputations. Due to small Ns, those experiencing five or more adversities are grouped

Descriptive statistics for the imputed and complete case samples can be found in Table 1. For the imputed data, the mean number of adversities children experienced was 1.13 (*SE*: 0.02) and the mean number of MRBs was 2.94 (*SE*: 0.04). The distribution of adversity scores was positively skewed with a median of 1 (Figure 1). The frequency of each adversity and their association with total MRBs are shown in Table 2. The most frequently reported adversity was parent mental health problems or suicide attempt (36.8%) with parental separation (19.2%) and violence between parents (18.7%) the next most common. Sexual abuse was the least experienced, with a frequency of less than 1% (complete case results for comparison are shown in Table S5). The prevalence of each adversity was higher in the imputed sample compared to the complete case sample, with physical abuse being the only exception. Of the individual adversities, the strongest evidence for an association with MRBs was found for parental substance use and parental criminal conviction (Table 2). Emotional abuse was strongly (*r* > .5), positively correlated with physical abuse. Emotional and physical abuse had the most moderate (.3 > *r* < .5) correlations with other adversities. The majority of correlations were weak (*r* < .3) (Table S6). Risk behaviours involving drug use (alcohol, tobacco, cannabis and other drugs) were strongly, positively correlated with each other and antisocial behaviour had multiple moderate correlations with other risk behaviours. Physical inactivity and excessive TV viewing had consistently weak correlations with other risk behaviours (Table S7). Correlations between individual adversities and risk behaviours were weak apart from some perfect, negative correlations between sexual abuse and some risk behaviours which can explained by the few instances (less than 1%) of sexual abuse in the data (Table S8). Of the individual risk behaviours, the strongest evidence for an association with total adversities was found for drug and tobacco use (Table S9).

**Table 2:** Exposure to adversities and associations between each adversity and total multiple health risk behaviours (MRBs) at 16 years old (imputed data *N* = 5,799)

Adversities	Descriptive statistics		Association with MRBs (adjusted)		
	*N*	Per cent	β	95% CI	*p*
Parent mental health problems or suicide	2,132	36.77	.40	0.25, 0.55	<.001
Parental separation	1,115	19.24	.72	0.52, 0.92	<.001
Violence between parents	1,084	18.69	.62	0.42, 0.82	<.001
Emotional abuse	922	15.90	.62	0.42, 0.83	<.001
Child experiences bullying	674	11.63	.22	−0.02, 0.45	.071
Parent substance use	521	8.98	.78	0.51, 1.05	<.001
Physical abuse	369	6.36	.58	0.29, 0.88	<.001
Parent criminal conviction	357	6.15	.74	0.45, 1.04	<.001
Sexual abuse	30	0.52	.38	−0.65, 1.40	.472

For definitions of each adversity, see Table S1. 750 imputed data sets. Associations between each adversity and MRBs modelled in linear regressions controlling for child sex, housing tenure, income, social class, maternal education, maternal age at birth and child IQ. CI, confidence interval, β, beta.

### Mediation results

**Figure 2:**
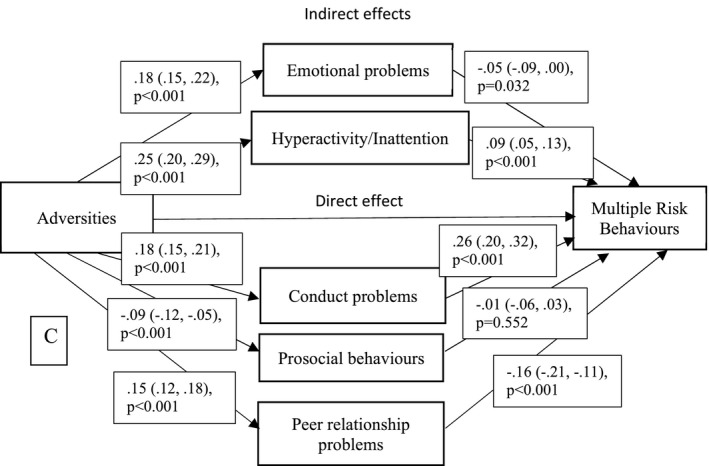
Directed acyclic graph (DAG) of mediation model. Notes: ‘C’ covariates: child sex, maternal education, income, social class, housing tenure, child IQ, maternal age at birth. SDQ, Strengths and Difficulties Questionnaire; MRBs, multiple risk behaviours. Adversities were measured from 0 to 9 years of age, SDQ at 12 years and MRBs at 16 years

We found a positive association between childhood adversities and adolescent MRBs; with each additional adversity being associated with a 0.25 increase in total MRBs (β .25, 95% CI 0.20, 0.31, *p* < .001; Table 3). We found robust evidence of indirect effects via peer relationship problems (indirect effect β −.02, 95% CI −0.03, −0.02, *p* < .001) such that each additional point on the peer relationship problems scale was associated with a –0.02 point decrease in the adversities‐MRB association. There was also robust evidence of mediation through hyperactivity/inattention (β .02, 95% CI 0.01, 0.03, *p* < .001) and conduct problems (β .05, 95% CI 0.03, 0.06, *p* < .001). Both hyperactivity/inattention and conduct problems had positive coefficients, indicating that increased levels of these problems were associated with an increase in the adversities‐MRB association. We found weaker evidence of an indirect effect via emotional symptoms (β −.01, 95% CI −0.02, −0.001, *p* = .037). Both peer relationship problems and emotional symptoms had negative coefficients, indicating that increased levels of these problems were associated with ameliorating the association between adversities and MRBs. We found no clear evidence of an indirect effect via prosocial behaviours (β .001, 95% CI −0.002, 0.005, *p* = .560). Adjusting for covariance between SDQ subscales did not alter results meaningfully (Table 3). Total, direct and indirect effects were broadly consistent in terms of direction and magnitude across imputed and complete case samples. Removing physical inactivity and excessive TV viewing from analysis did not meaningfully affect the direct effect or coefficients of paths between SDQ subscales and the alternate MRB construct (Figure S3). Adding an earlier measure of SDQ at age 9 as an additional potential confounder slightly weakened the mediating effects of SDQ at age 12 on the adversity‐MRB association but did not affect the direction of these associations (Figure S4). There was no evidence of an interaction between sex and total adversities in predicting total MRBs (Table S11) (Figure 2).

**Table 3:** Associations between adversities and multiple health risk behaviours through mediating pathways of strength and difficulties questionnaire subscales

	Adversities (unadjusted)		Adversities (adjusted)	
	β (95% CI)	*p*‐Value	β (95% CI)	*p*‐Value
Total effect	.32 (0.27, 0.37)	<.001	.29 (0.23, 0.35)	<.001
Direct effect	.28 (0.22, 0.33)	<.001	.25 (0.20, 0.31)	<.001
Specific indirect effects				
SDQ – Hyperactivity	.02 (0.01, 0.03)	<.001	.02 (0.01, 0.03)	<.001
SDQ – Emotional	−.01 (−0.02, 0.00)	.030	−.01 (−0.02, 0.00)	.037
SDQ – Conduct	.05 (0.04, 0.07)	<.001	.05 (0.03, 0.06)	<.001
SDQ – Peer	−.03 (−0.04, −0.02)	<.001	−.02 (−0.03, −0.02)	<.001
SDQ – Prosocial	.001 (−0.003, 0.004)	.907	.001 (0.00, 0.01)	.560

750 imputed data sets. Associations between adversities and MRBs modelled in structural equation modelling. Adjusted results controlling for child sex, housing tenure, income, social class, maternal education, maternal age at birth and child IQ. CI, confidence interval; β, beta; SDQ, strengths and difficulties questionnaire.

We calculated the proportion of effect mediated through the SDQ subscales. The direct effect from adversities to MRBs accounted for 65% of the total effect. The indirect effect through the Conduct Problems subscale accounted for 16.3% of the total effect followed by the Peer Relationship Problems subscale at 8.2% (Hyperactivity/Inattention 7.5%, Emotional Symptoms 3.0% and Prosocial 0.4%), with the total indirect effect accounting for 35% of the total effect.

## Discussion

This study investigated whether dimensions of child psychopathology act as mediators between number of adversities and MRBs. In line with existing studies (Bellis, Hughes, Leckenby, Jones, et al., 2014; Bellis, Hughes, Leckenby, Perkins, et al., 2014; Hughes et al., 2017), robust evidence was found for a positive association between total numbers of adversities and MRBs, with each additional adversity conferring a 0.25 increase in MRBs. In addition, we found robust evidence for mediation of effects via conduct problems, hyperactivity/inattention and peer relationship problems as measured by SDQ subscales. Findings suggest that higher levels of conduct problems and hyperactivity/inattention were associated with an increase in the adversities‐MRB association and higher levels of peer relationship problems were associated with a reduction in the adversities‐MRB association.

Children experiencing adversities are at high risk of developing mental and behavioural disorders (Scully, Mclaughlin, & Fitzgerald, 2020), and findings in this study suggest that high levels of conduct problems and hyperactivity/inattention were associated with an increase in the adversities‐MRB association. In support of this, a review and meta‐analysis of long‐term outcomes of attention deficit hyperactivity disorder (ADHD) and conduct disorders found associations with substance use disorders and criminality (Erskine et al., 2016), although no mechanisms were posited to explain these associations. Core symptoms of both disorders relating to impulsivity or distractibility may explain progression to MRBs. The CP subscale accounted for a larger percentage of the total mediating effect than the hyperactivity/inattention subscale (16.3% vs. 7.5%). This may be due to symptoms of CP such as aggressive or destructive behaviour and rule breaking being more directly related to initiation of MRBs than symptoms of hyperactivity/inattention. CP and ADHD symptoms independently contributed as mediating pathways and are both worthy of preventative efforts. Early interventions for children who have experienced adversities could include evidence‐based approaches known to prevent the onset or progression of CPs and hyperactivity/inattention to interrupt the progression to MRBs in adolescence. Parent training programmes have shown promise in alleviating ADHD symptoms in children (Fabiano et al., 2009) as well as co‐occurring oppositional and noncompliant behaviour (Bor, Sanders, & Markie‐Dadds, 2002).

There was a negative mediating effect of peer relationship problems on the relationship between adversities and MRBs, meaning, perhaps counter‐intuitively, higher scores on the peer relationship problems subscale were associated with a reduction in the adversities‐MRB association. Investigating a and b paths, adversity was positively associated with peer relationship problems which was expected, but surprisingly peer relationship problems were associated with fewer MRBs, which is why the overall indirect effect was negative. Children who experience adversities generally undergo higher levels of peer rejection experiences (Leve, Fisher, & Degarmo, 2007; Mccloskey & Stuewig, 2001) and have lower levels of peer likability, friendship and acceptance (Shen, 2009; Tung, Noroña, & Lee, 2019). Difficulties forming peer relationships may result in reduced opportunities to engage in MRBs with peers, and thus fewer MRBs. This is supported by evidence of associations between peer acceptance and adolescent substance use (Allen, Chango, Szwedo, Schad, & Marston, 2012). The peer relationship problems subscale summarises peer rejection experiences; therefore, our findings support these earlier studies and suggest children who have experienced adversities may need extra support to form peer relationships. A recent study of youth at risk of maltreatment found that the majority associated with prosocial peer groups and were less likely to engage in risk behaviours. The youth who socialised with severely antisocial peer groups were more likely to engage in adolescent risk behaviours (Yoon, 2020). Interventions to support children who have experienced adversities to form positive relationships with prosocial peers therefore may be protective for future multiple risk behaviours. Peer mentoring of maltreated children could be a viable approach to achieve this, and such interventions among maltreated preschool children result in improvements in social skills and a reduction in problem behaviours (Fantuzzo et al., 1996). Furthermore, peer mentoring of children in schools has been found to be an effective strategy to promote health behaviour change (Petosa & Smith, 2014). However, we need more research to fully understand the complex nuances around peer relationships and engaging in risky behaviours among children who have experienced adversities.

Although those with severe symptoms of ADHD and CD may benefit from intervention or prevention strategies to reduce risk of MRBs if they also have experienced adversity, our findings show that overall ADHD and CD symptoms only explain <25% of the association between adversities and MRBs. This means that interventions to prevent behavioural and emotional problems in childhood and early adolescence would not mitigate the majority of the risk conferred by adversities. Further research is needed to understand this direct link and what other interventions could reduce the progression from adversities to MRBs. Efforts should be focused on preventing adversities occurring in order to reduce engagement in risky behaviours in adolescence, as well as long‐term negative health impacts. Future research should also investigate other potential mediating pathways. There have been calls for adversity‐informed practice (i.e. practice that takes into account early‐life experiences in a holistic approach to the individual) in public services including in social care, education sector and the criminal justice system. There is a body of evidence that suggests that such an approach can prevent adversities and their downstream effects (Hughes et al., 2014). This joined‐up approach across public services is being implemented by Public Health Wales with the ‘Together for Mental Health’ strategy (Welsh Government, 2012).

### Strengths and limitations

The study is the first to explore whether broad domains of child psychopathology mediate the relationships between adversities and a wide range of adolescent MRBs. This is a strength compared to other studies that focus on a limited number of psychopathology domains and relatively few MRBs (Kim et al., 2018; Wardell et al., 2016). We had detailed information on a large sample of ˜6,000 young people followed longitudinally. The adversities included in our study were assessed across childhood from 0 to 9 years, and we included confounders in our analysis and rich and varied auxiliary variables to inform our imputation. Data on 13 distinct MRBs in late adolescence supplied us with a wide coverage of MRBs. Limitations of our study include the fact that nonresponse and loss to follow‐up occurs more frequently among individuals who experience adversities, socioeconomic disadvantage, psychopathology and who engage in more risk behaviours, which may have biased our results towards the null. We were, however, well placed to address this by using multiple imputation to account for missing data based on a comprehensive model of auxiliary data. It is difficult to mitigate all sources of bias, and the ALSPAC cohort remains more socially advantaged than the general population. Additional limitations are that we only used parent reports of psychopathology, and validity of the SDQ may have been improved if child and teacher reports were also available. Adversities were assessed largely by parent report; therefore, adversities may be underreported by parents who want to avoid self‐incrimination. Our study did not examine chronicity or severity of adversity exposure. Further work is needed to untangle the relationship between the duration and severity of adversity and outcomes of interest. Total adversities and MRBs were measured using a count system which gave each adversity/risky behaviour equal weight, which does not take into account their unique contributions to the disease burden.

## Conclusion

We found adversities between 0 and 9 years were associated with an increased engagement in adolescent MRBs and evidence these associations were mediated by conduct problems, hyperactivity/inattention and peer relationship problems. Strategies should target children who have higher levels of conducts problems and hyperactivity/inattention, or aim to ameliorate the impacts of these mental health problems. Early prevention work with children who have experienced adversities to avoid these problems is also necessary. Having high levels of peer relationship problems appears to result in engaging in fewer MRBs; interventions to increase peer connectivity without increasing MRB risk are needed. Ultimately, a broad, multidisciplinary approach to prevent adversities from occurring in the first instance and to address resultant mental health problems early could reduce the risk of engagement in adolescent MRBs and prevent premature morbidity and mortality in later life.

## Supporting information


**Figure S1.** Participant flow chart.
**Figure S2.** Path diagram of complete case results.
**Figure S3.** Path diagram of sensitivity analysis (excluding physical inactivity and excessive TV viewing risk behaviours).
**Figure S4.** Path diagram of sensitivity analysis (including Strength and Difficulties questionnaire responses at age 9 as an additional potential confounder).
**Table S1.** Adversity definitions.
**Table S2.** The ALSPAC variables used to derive the adversity constructs.
**Table S3.** Multiple health risk behaviours (MRBs) definitions.
**Table S4.** Questions that populate subscales on the parent‐report version of the Strengths and Difficulties Questionnaire for 4‐17 year olds.
**Table S5.** Frequencies of adversities in complete case study sample (*N* = 1,348).
**Table S6.** Tetrachoric correlations between 9 adversities (*N* = 3,965).
**Table S7.** Tetrachoric correlations between 13 risk behaviours (*N* = 2,656).
**Table S8.** Tetrachoric correlations between 9 adversities and 13 risk behaviours (unimputed data, *N* = 1,824).
**Table S9.** Associations between each risk behaviour and total adversities (imputed data, *N* = 5,799).
**Table S10.** Missingness of adversities and risk behaviours (imputed data, *N* = 5,799).
**Table S11.** Associations between adversities, sex, an interaction between sex and adversities and risk behaviours.
