# Supplementary material for: Childhood psychopathology mediates associations between childhood adversities and multiple health risk behaviours in adolescence: analysis using the ALSPAC birth cohort
Source: J Child Psychol Psychiatry. 2021 Feb 22;62(9):1100–9. doi: 10.1111/jcpp.13379 (PMC8532527; doi:10.1111/jcpp.13379)
Supplement: Supplementary file 1 — Figure S1. Participant flow chart. Figure S2. Path diagram of complete case results. Figure S3. Path diagram of sensitivity analysis (excluding physical inactivity and excessive TV viewing risk behaviours). Figure S4. Path diagram of sensitivity analysis (including Strength and Difficulties questionnaire responses at age 9 as an additional potential confounder). Table S1. Adversity definitions. Table S2. The ALSPAC variables used to derive the adversity constructs. Table S3. Multiple health risk behaviours (MRBs) definitions. Table S4. Questions that populate subscales on the parent‐report version of the Strengths and Difficulties Questionnaire for 4‐17 year olds. Table S5. Frequencies of adversities in complete case study sample (N = 1,348). Table S6. Tetrachoric correlations between 9 adversities (N = 3,965). Table S7. Tetrachoric correlations between 13 risk behaviours (N = 2,656). Table S8. Tetrachoric correlations between 9 adversities and 13 risk behaviours (unimputed data, N = 1,824). Table S9. Associations between each risk behaviour and total adversities (imputed data, N = 5,799). Table S10. Missingness of adversities and risk behaviours (imputed data, N = 5,799). Table S11. Associations between adversities, sex, an interaction between sex and adversities and risk behaviours. [file JCPP-62-1100-s001.docx]

**Supporting information – Childhood psychopathology mediates associations between childhood adversities and multiple health risk behaviours in adolescence: analysis using the ALSPAC birth cohort – by Troy *et al*.**

## **Figure S1.** Participant flow chart.

Enrolled cohort singletons and first born twins alive at 1 year (n=13,978)

Mothers invited to complete SDQ when their child was 12 years old (n=10,792)

Complete case sample: Adversities, MRB & SDQ data (n=1,348)

Complete data on SDQ subscales: Imputation sample (n=5,799)

MRBs, multiple health risk behaviours. SDQ, Strength and Difficulties Questionnaire.

## **Figure S2.** Path diagram of complete case results.

-.01 (-.08, .06), p=0.763

.23 (.12, .33), p<0.001

.12 (.06, .18), p<0.001

-.04 (-.11, .04), p=0.321

.19 (.11, .27), p<0.001

-.07 (-.14, .00), p=0.058

.11 (.05, .18), p<0.001

.21 (.12, .31), p<0.001

.20 (.13, .28), p<0.001

Hyperactivity/Inattention

β 0.03, 95% CI 0.01, 0.04, p=0.002

Conduct problems

β 0.03, 95% CI 0.01, 0.04, p=0.007

Prosocial behaviours

β 0.001, 95% CI -0.004, 0.006, p=0.764

Indirect effects

Adversities

Multiple Health Risk Behaviours

Emotional problems

β -0.01, 95% CI -0.02, 0.01, p=0.335

Direct effect

β 0.18, 95% CI 0.10, 0.27, p<0.001

-.15 (-.22, -.07), p<0.001

C

Peer relationship problems

β -0.03, 95% CI -0.05, -0.01, p=0.002

β beta. CI confidence interval. C confounders. Adversities were measured from 0-9 years of age, SDQ at 12 years, and MRBs at 16 years. Notes: paths are adjusted for intermediate confounders: see main manuscript. Complete case sample *N*=1348.

**Figure S3.** Path diagram of sensitivity analysis (excluding physical inactivity and excessive TV viewing risk behaviours).

-.02 (-.06, .03), p=0.452

-.09 (-.12, -.05), p<0.001

.25 (.20, .29), p<0.001

.18 (.15, .21), p<0.001

.18 (.15, .22), p<0.001

Hyperactivity/Inattention

β 0.02, 95% CI 0.01, 0.03, p<0.001

Conduct problems

β 0.04, 95% CI 0.03, 0.06, p<0.001

.09 (.05, .12), p<0.001

-.06 (-.11, -.02), p=0.003

.25 (.18, .31), p<0.001

Indirect effects

Adversities

Multiple Health Risk Behaviours

Emotional problems

β -0.01, 95% CI -0.02, -0.01, p=0.005

Direct effect

β 0.25, 95% CI 0.20, 0.30, p<0.001

.15 (.12, .18), p<0.001

Prosocial behaviours

β 0.001, 95% CI -0.002, 0.005, p=0.462

C

-.16 (-.21, -.12), p<0.001

Peer relationship problems

β -0.02, 95% CI -0.03, -0.02, p<0.001

β beta. CI confidence interval. C confounders. Adversities were measured from 0-9 years of age, SDQ at 12 years, and MRBs at 16 years. Notes: paths are adjusted for intermediate confounders: see main manuscript. Imputed sample, *N*=5799.

**Figure S4.** Path diagram of sensitivity analysis (including Strength and Difficulties questionnaire responses at age 9 as an additional potential confounder).

-.03 (-.08, .03), p=0.324

-.02 (-.05, .01), p=0.143

Prosocial behaviours

β 0.001, 95% CI -0.001, 0.002, p=0.443

.06 (.03, .09), p<0.001

.08 (.05, .11), p<0.001

.05 (.02, .07), p<0.001

-.05 (-.10, .00), p=0.073

.12 (.07, .17), p<0.001

.17 (.09, .24), p<0.001

Hyperactivity/Inattention

β 0.007, 95% CI 0.002, 0.012, p=0.004

Conduct problems

β 0.008, 95% CI 0.002, 0.014, p=0.004

Indirect effects

Adversities

Multiple Health Risk Behaviours

Emotional problems

β -0.004, 95% CI -0.008, 0.001, p=0.093

Direct effect

β 0.25, 95% CI 0.19, 0.31, p<0.001

-.13 (-.19, -.07), p<0.001

.04 (.01, .06), p=0.005

C

Peer relationship problems

β -0.005, 95% CI -0.009, -0.001, p=0.020

## β beta. CI confidence interval. C confounders. Adversities were measured from 0-9 years of age, SDQ at 12 years, and MRBs at 16 years. Notes: paths are adjusted for intermediate confounders: see main manuscript. Imputed sample, *N*=4679.

## **Table S1.** Adversity definitions.

| **Adversities** | **Definition** | **Number of questions** | **Age range of questions asked** | **Respondents** |
| --- | --- | --- | --- | --- |
| Sexual abuse | Was the child sexually abused | 7 | 18 months to age 9 | Mother |
| Physical abuse | Whether physically cruel to child | 31 | 8 weeks to 9 years | Mother and Partner |
| Emotional abuse | Whether or not mum/partner had been emotionally cruel to the child | 32 | 8 months to 9 years | Mother and Partner |
| Parent substance use | Daily use of cannabis or any use of other drugs. Or, alcohol problem by self-reported problematic use, and saw a doctor because of it | 62 | 8 weeks to 9 years | Mother and Partner |
| Parent mental health problems or suicide attempt | Depression scores (EPDS>12) and medication, presence of schizophrenia, bulimia, anorexia or attempted suicide. | 57 | 8 weeks to 9 years | Mother and Partner |
| Violence between parents | Parent experienced physical cruelty from partner, or displayed (specific types) of violence towards partner | 43 | 8 weeks to 9 years | Mother and Partner |
| Parental separation | Parents divorced or separated. Degree to which this impacted on the child. | 32 | 8 weeks to 9 years | Mother and Partner |
| Bullying | Child bullied | 6 | 8 years to8.5 years | Child |
| Parent criminal conviction | Parent convicted of offence | 18 | 8 weeks to 9 years | Mother and Partner |

Notes: EPDS Edinburgh Postnatal Depression Scale. Several of the adversities included questions about how much the adversity had impacted on the parent or child.

**Table S2.** The ALSPAC variables used to derive the adversity constructs.

| **Description** | **reported** | **retrospective** | **ACE** | **Age reported** | **Start time period** | **End time period** |
| --- | --- | --- | --- | --- | --- | --- |
| Frequency child has been bullied | Child | no | bullying | 97m | 8 | 8 |
| Personal belongings stolen | Child | no | bullying | 8.5yrs | 8.5 | 8.5 |
| Threatened/blackmailed | Child | no | bullying | 8.5yrs | 8.5 | 8.5 |
| Hit/beaten up | Child | no | bullying | 8.5yrs | 8.5 | 8.5 |
| Got to do something didn't want to | Child | no | bullying | 8.5yrs | 8.5 | 8.5 |
| Told lies about | Child | no | bullying | 8.5yrs | 8.5 | 8.5 |
| Personal belongings stolen | Child | no | bullying | 10+yrs | 10 | 10 |
| Threatened/blackmailed | Child | no | bullying | 10+yrs | 10 | 10 |
| Hit/beaten up | Child | no | bullying | 10+yrs | 10 | 10 |
| Got to do something didn't want to | Child | no | bullying | 10+yrs | 10 | 10 |
| Told lies about | Child | no | bullying | 10+yrs | 10 | 10 |
| Someone threatened/blackmailed teenager | Child | no | bullying | 12.5yrs | 12.5 | 12.5 |
| Friends tried to get teenager to do things didnt want to do | Child | no | bullying | 12.5yrs | 12.5 | 12.5 |
| Friends told lies about teenager | Child | no | bullying | 12.5yrs | 12.5 | 12.5 |
| YPs friends put YP down in front of others | Child | no | bullying | 15.5yrs | 15.5 | 15.5 |
| YPs friends put pressure on YP to do things they don't want to do | Child | no | bullying | 15.5yrs | 15.5 | 15.5 |
| During the last school year YP has been upset by name calling/exclusion from groups or bullying | Child | no | bullying | year 11 | 16 | 16 |
| YP has been directly bullied in last 6 mths | Child | no | bullying | 17.5yrs | 17.5 | 17.5 |
| YP has been relationally bullied in last 6 mths | Child | no | bullying | 17.5yrs | 17.5 | 17.5 |
| PTNR was EMOT cruel to CH since PREG | Parent | no | emotional abuse | 18w gest | -1 | -1 |
| PTNR EMOT cruel to CHDR >CH born | Parent | no | emotional abuse | 8m | 0 | 0.67 |
| MUM EMOT cruel to CHDR >CH born | Parent | no | emotional abuse | 8m | 0 | 0.67 |
| Ptnr Emotionally Cruel To Children | Parent | no | emotional abuse | 8m | 0 | 0.67 |
| Self Emotionally Cruel To Children | Parent | no | emotional abuse | 8m | 0 | 0.67 |
| Frequency adult in family shouted at respondent before age of 11 | Child | yes | emotional abuse | 22yrs | 0 | 11 |
| Frequency adult in family said hurtful or insulting things to respondent before age of 11 | Child | yes | emotional abuse | 22yrs | 0 | 11 |
| When growing up respondent felt that someone in their family hated them | Child | yes | emotional abuse | 23yrs | 0 | 16 |
| PTNR EMOT cruel to CH | Parent | no | emotional abuse | 8w | 0.16 | 0.16 |
| Partner emotionally cruel to children >CH8MTHs | Parent | no | emotional abuse | 1yrs9m | 0.67 | 2 |
| Mum emotionally cruel to children >CH18MTHs | Parent | no | emotional abuse | 1yrs9m | 0.67 | 2 |
| Partner Emotionally Cruel To Child | Parent | no | emotional abuse | 1yrs9m | 0.67 | 2 |
| Self Emotionally Cruel To Child | Parent | no | emotional abuse | 1yrs9m | 0.67 | 2 |
| Whether partner was emotionally cruel to children since study child was 18 months old and effect this had | Parent | no | emotional abuse | 2yrs9m | 1.5 | 3 |
| Whether mum was emotionally cruel to children since study child was 18 months old and effect this had | Parent | no | emotional abuse | 2yrs9m | 1.5 | 3 |
| Partner's partner was emotionally cruel to their children since study child was 18 months old | Parent | no | emotional abuse | 2yrs9m | 1.5 | 3 |
| Partner was emotionally cruel to their children since study child was 18 months old | Parent | no | emotional abuse | 2yrs9m | 1.5 | 3 |
| PTR Emotional Cruel to CDRN> CH 30 MTHs | Parent | no | emotional abuse | 3yrs11m | 2.5 | 4 |
| MUM Emotional Cruel to CDRN> CH 30 MTHs | Parent | no | emotional abuse | 3yrs11m | 2.5 | 4 |
| Partner's emotional cruelty towards children affected partner since child was 2.5 years old | Parent | no | emotional abuse | 3yrs11m | 2.5 | 4 |
| Partner's emotional cruelty towards children affected partner since child was 2.5 years old | Parent | no | emotional abuse | 3yrs11m | 2.5 | 4 |
| Mothers partner was emotionally cruel to children in past year | Parent | no | emotional abuse | 5yrs1m | 4 | 5 |
| Mother was emotionally cruel to children in past year | Parent | no | emotional abuse | 5yrs1m | 4 | 5 |
| Respondent's assessment of how much their partner being emotionally cruel to the children in the last year has affected them | Parent | no | emotional abuse | 5yrs1m | 4 | 5 |
| Respondent's assessment of how much being emotionally cruel to their children in the last year has affected them | Parent | no | emotional abuse | 5yrs1m | 4 | 5 |
| Respondent's partner was emotionally cruel to respondent's children since study child's 5th birthday | Parent | no | emotional abuse | 6yrs1m | 5 | 6 |
| Respondent was emotionally cruel to their children since study child's 5th birthday | Parent | no | emotional abuse | 6yrs1m | 5 | 6 |
| Respondent's assessment of how much partner's emotional cruelty to children since study child's 5th birthday has affected them | Parent | no | emotional abuse | 6yrs1m | 5 | 6 |
| Respondent's assessment of how much being emotionally cruel to children since study child's 5th birthday has affected them | Parent | no | emotional abuse | 6yrs1m | 5 | 6 |
| Mother's husband/partner was emotionally cruel to her children since the study child's 6th birthday | Parent | yes | emotional abuse | 9yrs2m | 6 | 7 |
| Mother was emotionally cruel to her children since the study child's 6th birthday | Parent | yes | emotional abuse | 9yrs2m | 6 | 7 |
| Father's wife/partner was emotionally cruel to his children since the study child's 6th birthday | Parent | yes | emotional abuse | 9yrs2m | 6 | 7 |
| Father was emotionally cruel to his children since the study child's 6th birthday | Parent | yes | emotional abuse | 9yrs2m | 6 | 7 |
| Mother's husband/partner was emotionally cruel to her children since the study child's 6th birthday | Parent | no | emotional abuse | 9yrs2m | 8 | 9 |
| Mother was emotionally cruel to her children since the study child's 6th birthday | Parent | no | emotional abuse | 9yrs2m | 8 | 9 |
| Father's wife/partner was emotionally cruel to his children since the study child's 6th birthday | Parent | no | emotional abuse | 9yrs2m | 8 | 9 |
| Father was emotionally cruel to his children since the study child's 6th birthday | Parent | no | emotional abuse | 9yrs2m | 8 | 9 |
| Respondent's wife/partner has been emotionally cruel to their children since the study child's 9th birthday | Parent | yes | emotional abuse | 11yrs2m | 9 | 10 |
| Respondent has been emotionally cruel to their children since the study child's 9th birthday | Parent | yes | emotional abuse | 11yrs2m | 9 | 10 |
| Respondent's husband/partner has been emotionally cruel to their children since study child's 9th birthday | Parent | yes | emotional abuse | 11yrs2m | 9 | 10 |
| Respondent has been emotionally cruel to their children since study child's 9th birthday | Parent | yes | emotional abuse | 11yrs2m | 9 | 10 |
| Respondent's wife/partner has been emotionally cruel to their children since the study child's 9th birthday | Parent | no | emotional abuse | 11yrs2m | 11 | 11 |
| Respondent has been emotionally cruel to their children since the study child's 9th birthday | Parent | no | emotional abuse | 11yrs2m | 11 | 11 |
| Respondent's husband/partner has been emotionally cruel to their children since study child's 9th birthday | Parent | no | emotional abuse | 11yrs2m | 11 | 11 |
| Respondent has been emotionally cruel to their children since study child's 9th birthday | Parent | no | emotional abuse | 11yrs2m | 11 | 11 |
| Frequency adult in family shouted at respondent between ages of 11 and 17 | Child | yes | emotional abuse | 22yrs | 11 | 17 |
| Frequency adult in family said hurtful or insulting things to respondent between ages of 11 and 17 | Child | yes | emotional abuse | 22yrs | 11 | 17 |
| Respondent's partner was emotionally cruel to respondent's children in last year | Parent | no | emotional abuse | 2010 | 17.5 | 18.5 |
| Respondent was emotionally cruel to own children in last year | Parent | no | emotional abuse | 2010 | 17.5 | 18.5 |
| In last year, partner was emotionally cruel to respondent's children | Parent | no | emotional abuse | 2011-2013 | 19.5 | 20.5 |
| In last year, was emotionally cruel to own children | Parent | no | emotional abuse | 2011-2013 | 19.5 | 20.5 |
| Edinburgh Postnatal Depression Score | Parent | no | mental health problems | 18w gest | -1 | -1 |
| EPDS in YP | Parent | no | mental health problems | 32w gest | -1 | -1 |
| EPDS Score I | Parent | no | mental health problems | 18w gest | -1 | -1 |
| Medication for anxiety this PREG | Parent | no | mental health problems | 18w gest | -1 | -1 |
| Medication for anxiety in 1st 3 months | Parent | no | mental health problems | 18w gest | -1 | -1 |
| Medication for depression this PREG | Parent | no | mental health problems | 18w gest | -1 | -1 |
| Medication for depression in 1st 3 months | Parent | no | mental health problems | 18w gest | -1 | -1 |
| Attempted suicide since PREG | Parent | no | mental health problems | 18w gest | -1 | -1 |
| MEDTN for anxiety in last 3MTHS | Parent | no | mental health problems | 32w gest | -1 | -1 |
| MEDTN for depression in last 3MTHS | Parent | no | mental health problems | 32w gest | -1 | -1 |
| Had bulimia | Parent | no | mental health problems | 15w gest | -1 | -1 |
| Had schizophrenia | Parent | no | mental health problems | 15w gest | -1 | -1 |
| Had anorexia nervosa | Parent | no | mental health problems | 15w gest | -1 | -1 |
| Had bulimia | Parent | no | mental health problems | 12w gest | -1 | -1 |
| Had schizophrenia | Parent | no | mental health problems | 12w gest | -1 | -1 |
| Had anorexia nervosa | Parent | no | mental health problems | 12w gest | -1 | -1 |
| Attempted suicide since PTNR PREG | Parent | no | mental health problems | 18w gest | -1 | -1 |
| FREQ of anti-depressant use since birth | Parent | no | mental health problems | 8w | 0 | 0.16 |
| Anti-depressant use since CH born | Parent | no | mental health problems | 8m | 0 | 0.67 |
| Attempted suicide > CH born | Parent | no | mental health problems | 8m | 0 | 0.67 |
| PTNR had schizophrenia >CH born | Parent | no | mental health problems | 8m | 0 | 0.67 |
| Used Pills for Depression Since Baby Born | Parent | no | mental health problems | 8m | 0 | 0.67 |
| Attempted Suicide Since Baby Born | Parent | no | mental health problems | 8m | 0 | 0.67 |
| EPDS | Parent | no | mental health problems | 8w | 0.16 | 0.16 |
| EPDS Score I | Parent | no | mental health problems | 8w | 0.16 | 0.16 |
| Attempted suicide since MID PREG | Parent | no | mental health problems | 8w | -1 | 0.16 |
| Attempted suicide since MID PREG | Parent | no | mental health problems | 8w | -1 | 0.16 |
| Edinburgh Post-natal Depression Score | Parent | no | mental health problems | 8m | 0.67 | 0.67 |
| Edinburgh Post-natal Depression Score | Parent | no | mental health problems | 8m | 0.67 | 0.67 |
| Mum had depression pills >CH8MTHs | Parent | no | mental health problems | 1yrs9m | 0.67 | 2 |
| Mum attempted suicide >CH8MTHs | Parent | no | mental health problems | 1yrs9m | 0.67 | 2 |
| Partner had schizophrenia >CH8MTHs | Parent | no | mental health problems | 1yrs9m | 0.67 | 2 |
| Anxiety Since Child > 8 Months | Parent | no | mental health problems | 1yrs9m | 0.67 | 2 |
| Taken Antidepressants CH > 8 Months | Parent | no | mental health problems | 1yrs9m | 0.67 | 2 |
| Frequency Mum has taken pills for depression since study child was 18 months old | Parent | no | mental health problems | 2yrs9m | 1.5 | 3 |
| Whether mum attempted suicide since study child was 18 months old and effect this had | Parent | no | mental health problems | 2yrs9m | 1.5 | 3 |
| Partner had schizophrenia since study child was 18 months old | Parent | no | mental health problems | 2yrs9m | 1.5 | 3 |
| Partner attempted suicide since study child was 18 months old | Parent | no | mental health problems | 2yrs9m | 1.5 | 3 |
| Edinburgh Post-natal Depression Score | Parent | no | mental health problems | 1yrs9m | 2 | 2 |
| EPDS Score I | Parent | no | mental health problems | 1yrs9m | 2 | 2 |
| Attempted Suicide | Parent | no | mental health problems | 1yrs9m | 2 | 2 |
| MUM Attempted Suicide> CH 30 MTHs | Parent | no | mental health problems | 3yrs11m | 2.5 | 4 |
| Degree to which attempted suicide affected partner since child was 2.5 years old | Parent | no | mental health problems | 3yrs11m | 2.5 | 4 |
| Edinburgh postnatal depression scale score (complete cases) | Parent | no | mental health problems | 2yrs9m | 3 | 3 |
| MUM took Depression Pills >1 YR | Parent | no | mental health problems | 3yrs11m | 3 | 4 |
| Partner had Schizophrenia> 1 YR | Parent | no | mental health problems | 3yrs11m | 3 | 4 |
| Frequency partner has taken pills for depression in the past year | Parent | no | mental health problems | 3yrs11m | 3 | 4 |
| Mother had schizophrenia in past year | Parent | no | mental health problems | 5yrs1m | 4 | 5 |
| Frequency mother had pills for depression in past year | Parent | no | mental health problems | 5yrs1m | 4 | 5 |
| Mother attempted suicide in past year | Parent | no | mental health problems | 5yrs1m | 4 | 5 |
| Respondent had schizophrenia in the past year | Parent | no | mental health problems | 5yrs1m | 4 | 5 |
| Frequency in the past year respondent has taken pills for depression | Parent | no | mental health problems | 5yrs1m | 4 | 5 |
| Respondent's assessment of how much attempting suicide in the last year has affected them | Parent | no | mental health problems | 5yrs1m | 4 | 5 |
| Respondent has had/continued to have schizophrenia since study child's 5th birthday | Parent | no | mental health problems | 6yrs1m | 5 | 6 |
| Frequency respondent has taken pills for depression since study child's 5th birthday | Parent | no | mental health problems | 6yrs1m | 5 | 6 |
| Respondent attempted suicide since study child's 5th birthday | Parent | no | mental health problems | 6yrs1m | 5 | 6 |
| Respondent's partner has had schizophrenia since study child was 5 years old | Parent | no | mental health problems | 6yrs1m | 5 | 6 |
| Respondent has suffered from schizophrenia since child's 5th birthday | Parent | no | mental health problems | 6yrs1m | 5 | 6 |
| Respondent has taken pills for depression since child's 5th birthday | Parent | no | mental health problems | 6yrs1m | 5 | 6 |
| Respondent's assessment of how much attempting suicide since study child's 5th birthday has affected them | Parent | no | mental health problems | 6yrs1m | 5 | 6 |
| Respondent's partner has had schizophrenia since study child was 5 | Parent | no | mental health problems | 6yrs1m | 5 | 6 |
| Mother attempted suicide since the study child's 6th birthday | Parent | yes | mental health problems | 9yrs2m | 6 | 7 |
| Father attempted suicide since the study child's 6th birthday | Parent | yes | mental health problems | 9yrs2m | 6 | 7 |
| Father has had schizophrenia in last 3 years | Parent | no | mental health problems | 9yrs2m | 6 | 9 |
| Mother has had schizophrenia in last 3 years | Parent | no | mental health problems | 9yrs2m | 6 | 9 |
| Frequency mother has taken pills for depression in last 2 years | Parent | no | mental health problems | 9yrs2m | 7 | 9 |
| Husband/partner has had schizophrenia, in last 2 years | Parent | no | mental health problems | 9yrs2m | 7 | 9 |
| Frequency father has taken pills for depression in last 2 years | Parent | no | mental health problems | 9yrs2m | 7 | 9 |
| Wife/partner has had schizophrenia, in last 2 years | Parent | no | mental health problems | 9yrs2m | 7 | 9 |
| Mother has ever had bulimia | Parent | no | mental health problems | 8yrs1m | 8 | 8 |
| Mother has ever had schizophrenia | Parent | no | mental health problems | 8yrs1m | 8 | 8 |
| Mother has ever had anorexia nervosa | Parent | no | mental health problems | 8yrs1m | 8 | 8 |
| Respondent has ever had bulimia | Parent | no | mental health problems | 8yrs1m | 8 | 8 |
| Respondent has ever had schizophrenia | Parent | no | mental health problems | 8yrs1m | 8 | 8 |
| Respondent has ever had anorexia nervosa | Parent | no | mental health problems | 8yrs1m | 8 | 8 |
| Mother attempted suicide since the study child's 6th birthday | Parent | no | mental health problems | 9yrs2m | 8 | 9 |
| Father attempted suicide since the study child's 6th birthday | Parent | no | mental health problems | 9yrs2m | 8 | 9 |
| Respondent has taken medicines for depression in the past 12 months | Parent | no | mental health problems | 10yrs2m | 9 | 10 |
| Respondent has taken medicines for anxiety/nerves in the past 12 months | Parent | no | mental health problems | 10yrs2m | 9 | 10 |
| Mother used medicine in last 12 months for depression | Parent | no | mental health problems | 10yrs2m | 9 | 10 |
| Mother used medicine in last 12 months for anxiety/nerves | Parent | no | mental health problems | 10yrs2m | 9 | 10 |
| Respondent attempted suicide since the study child's 9th birthday | Parent | yes | mental health problems | 11yrs2m | 9 | 10 |
| Respondent attempted suicide since the study child's 9th birthday | Parent | yes | mental health problems | 11yrs2m | 9 | 10 |
| Respondent attempted suicide since the study child's 9th birthday | Parent | no | mental health problems | 11yrs2m | 11 | 11 |
| Respondent attempted suicide since the study child's 9th birthday | Parent | no | mental health problems | 11yrs2m | 11 | 11 |
| Partner has had schizophrenia in the last two years | Parent | no | mental health problems | 12yrs1m | 10 | 12 |
| Partner's partner had schizophrenia in the last 2 years | Parent | no | mental health problems | 12yrs1m | 10 | 12 |
| Mother has had schizophrenia in last 2 years | Parent | no | mental health problems | 12yrs1m | 10 | 12 |
| Mother's partner has had schizophrenia since study child's 10th birthday | Parent | no | mental health problems | 12yrs1m | 10 | 12 |
| Respondent has ever had bulimia | Parent | no | mental health problems | 11yrs2m | 11 | 11 |
| Respondent has ever had schizophrenia | Parent | no | mental health problems | 11yrs2m | 11 | 11 |
| Respondent has ever had anorexia nervosa | Parent | no | mental health problems | 11yrs2m | 11 | 11 |
| Respondent has ever had bulimia | Parent | no | mental health problems | 11yrs2m | 11 | 11 |
| Respondent has ever had schizophrenia | Parent | no | mental health problems | 11yrs2m | 11 | 11 |
| Respondent has ever had anorexia nervosa | Parent | no | mental health problems | 11yrs2m | 11 | 11 |
| Partner has used medicine for depression in the last 12 months | Parent | no | mental health problems | 12yrs1m | 11 | 12 |
| Partner has used medicine for anxiety or nerves in the last 12 months | Parent | no | mental health problems | 12yrs1m | 11 | 12 |
| Mother has taken medication for depression in the past 12 months | Parent | no | mental health problems | 12yrs1m | 11 | 12 |
| Mother has taken medication for anxiety or nerves in the past 12 months | Parent | no | mental health problems | 12yrs1m | 11 | 12 |
| YP's mum has hurt themselves on purpose | Child | no | mental health problems | 16yrs | 16 | 16 |
| YP's dad has hurt themselves on purpose | Child | no | mental health problems | 16yrs | 16 | 16 |
| Frequency respondent taken pills for depression in last two years | Parent | no | mental health problems | 2010 | 16.5 | 18.5 |
| Respondent attempted suicide in last year | Parent | no | mental health problems | 2010 | 17.5 | 18.5 |
| EPDS Total score | Parent | no | mental health problems | 2010 | 18.5 | 18.5 |
| Respondent ever admitted to hospital for psychiatric or mental health problems | Parent | no | mental health problems | 2010 | 18.5 | 18.5 |
| Study child's biological father ever admitted to hospital for psychiatric or mental health problems | Parent | no | mental health problems | 2010 | 18.5 | 18.5 |
| Respondent has ever had an illness that included hearing voices or seeing things that were not there | Parent | no | mental health problems | 2010 | 18.5 | 18.5 |
| Respondent ever had an illness with paranoid delusions or developed unusual false beliefs | Parent | no | mental health problems | 2010 | 18.5 | 18.5 |
| Study child's biological father ever had an illness with paranoid delusions or developed unusual false beliefs | Parent | no | mental health problems | 2010 | 18.5 | 18.5 |
| Respondent ever suffered from schizophrenia | Parent | no | mental health problems | 2010 | 18.5 | 18.5 |
| Study child's biological father ever suffered from schizophrenia | Parent | no | mental health problems | 2010 | 18.5 | 18.5 |
| Respondent ever suffered from a manic illness | Parent | no | mental health problems | 2010 | 18.5 | 18.5 |
| Study child's biological father ever suffered from a manic illness | Parent | no | mental health problems | 2010 | 18.5 | 18.5 |
| Pills for depression | Parent | no | mental health problems | 2011-2013 | 18.5 | 20.5 |
| In last year, attempted suicide | Parent | no | mental health problems | 2011-2013 | 19.5 | 20.5 |
| Convicted of an offence since PREG | Parent | no | parent convicted | 18w gest | -1 | -1 |
| Convicted of offence since PTNR PREG,Y/N | Parent | no | parent convicted | 18w gest | -1 | -1 |
| Convicted since MID PREG | Parent | no | parent convicted | 8w | 0.16 | 0.16 |
| Convicted since MID PREG, Y/N | Parent | no | parent convicted | 8w | 0.16 | 0.16 |
| Court conviction | Parent | no | parent convicted | 8m | 0.67 | 0.67 |
| Convicted of Offence Since Baby Born | Parent | no | parent convicted | 8m | 0.67 | 0.67 |
| Mum convicted of offence >CH8MTHs | Parent | no | parent convicted | 1yrs9m | 0.67 | 2 |
| Mum convicted of offence >CH8MTHs | Parent | no | parent convicted | 1yrs9m | 0.67 | 2 |
| Convicted of Offence Y/N | Parent | no | parent convicted | 1yrs9m | 0.67 | 2 |
| Whether mum was convicted of an offence since study child was 18 months old and effect this had | Parent | no | parent convicted | 2yrs9m | 1.5 | 3 |
| Partner was convicted of an offence since study child was 18 months old | Parent | no | parent convicted | 2yrs9m | 1.5 | 3 |
| MUM Convicted of Offence> CH 30 MTHs | Parent | no | parent convicted | 3yrs11m | 2.5 | 4 |
| Degree to which a criminal conviction affected partner since child was 2.5 years old | Parent | no | parent convicted | 3yrs11m | 2.5 | 4 |
| Mother was convicted of an offence in past year | Parent | no | parent convicted | 5yrs1m | 4 | 5 |
| Respondent's assessment of how much being convicted of an offence in the last year has affected them | Parent | no | parent convicted | 5yrs1m | 4 | 5 |
| Respondent convicted of an offence since study child's 5th birthday | Parent | no | parent convicted | 6yrs1m | 5 | 6 |
| Respondent's assessment of how much being convicted of an offence since study child's 5th birthday has affected them | Parent | no | parent convicted | 6yrs1m | 5 | 6 |
| Mother was convicted of an offence since the study child's 6th birthday | Parent | yes | parent convicted | 9yrs2m | 6 | 7 |
| Father was convicted of an offence since the study child's 6th birthday | Parent | yes | parent convicted | 9yrs2m | 6 | 7 |
| Mother was convicted of an offence since the study child's 6th birthday | Parent | no | parent convicted | 9yrs2m | 8 | 9 |
| Father was convicted of an offence since the study child's 6th birthday | Parent | no | parent convicted | 9yrs2m | 8 | 9 |
| Respondent has been convicted of an offence since the study child's 9th birthday | Parent | yes | parent convicted | 11yrs2m | 9 | 10 |
| Respondent was convicted of an offence since study child's 9th birthday | Parent | yes | parent convicted | 11yrs2m | 9 | 10 |
| Respondent has been convicted of an offence since the study child's 9th birthday | Parent | no | parent convicted | 11yrs2m | 11 | 11 |
| Respondent was convicted of an offence since study child's 9th birthday | Parent | no | parent convicted | 11yrs2m | 11 | 11 |
| Partner has been convicted of an offence other then speeding in the last year | Parent | no | parent convicted | 12yrs1m | 11 | 12 |
| Mother was convicted of an offence in the last year | Parent | no | parent convicted | 12yrs1m | 11 | 12 |
| Respondent was convicted of an offence in last year | Parent | no | parent convicted | 2010 | 17.5 | 18.5 |
| In last year, was convicted of an offence | Parent | no | parent convicted | 2011-2013 | 19.5 | 20.5 |
| Divorced since PREG | Parent | no | parental separation | 18w gest | -1 | -1 |
| Separated since PREG | Parent | no | parental separation | 18w gest | -1 | -1 |
| Divorced since PTNR PREG | Parent | no | parental separation | 18w gest | -1 | -1 |
| Separated since PTNR PREG | Parent | no | parental separation | 18w gest | -1 | -1 |
| Divorce >CH born | Parent | no | parental separation | 8m | 0 | 0.67 |
| Separation from PTNR >CH born | Parent | no | parental separation | 8m | 0 | 0.67 |
| Divorced Since Baby Born | Parent | no | parental separation | 8m | 0 | 0.67 |
| Separated Since Baby Born | Parent | no | parental separation | 8m | 0 | 0.67 |
| Divorced since MID PREG | Parent | no | parental separation | 8w | -1 | 0.16 |
| Separated since MID PREG | Parent | no | parental separation | 8w | -1 | 0.16 |
| Divorced since MID PREG | Parent | no | parental separation | 8w | -1 | 0.16 |
| You & PTNR separated since MID PREG | Parent | no | parental separation | 8w | -1 | 0.16 |
| Mum divorced >CH8MTHs | Parent | no | parental separation | 1yrs9m | 0.67 | 2 |
| Mum and partner separated >CH8MTHs | Parent | no | parental separation | 1yrs9m | 0.67 | 2 |
| Divorced | Parent | no | parental separation | 1yrs9m | 0.67 | 2 |
| Separated From Partner | Parent | no | parental separation | 1yrs9m | 0.67 | 2 |
| Whether mum got divorced since study child was 18 months old and effect this had | Parent | no | parental separation | 2yrs9m | 1.5 | 3 |
| Whether mum and partner separated since study child was 18 months old and effect this had | Parent | no | parental separation | 2yrs9m | 1.5 | 3 |
| Partner was divorced since study child was 18 months old | Parent | no | parental separation | 2yrs9m | 1.5 | 3 |
| Partner and partner's partner have separated since study child was 18 months old | Parent | no | parental separation | 2yrs9m | 1.5 | 3 |
| MUM Divorced> CH 30 MTHs | Parent | no | parental separation | 3yrs11m | 2.5 | 4 |
| MUM & PTR Separated> CH 30 MTHs | Parent | no | parental separation | 3yrs11m | 2.5 | 4 |
| Degree to which divorce affected partner since child was 2.5 years old | Parent | no | parental separation | 3yrs11m | 2.5 | 4 |
| Degree to which separation affected partner since child was 2.5 years old | Parent | no | parental separation | 3yrs11m | 2.5 | 4 |
| Mother was divorced in past year | Parent | no | parental separation | 5yrs1m | 4 | 5 |
| Mother and partner separated in past year | Parent | no | parental separation | 5yrs1m | 4 | 5 |
| Respondent's assessment of how much separating from their partner in the last year has affected them | Parent | no | parental separation | 5yrs1m | 4 | 5 |
| Respondent was divorced since study child's 5th birthday | Parent | no | parental separation | 6yrs1m | 5 | 6 |
| Respondent separated from partner since study child's 5th birthday | Parent | no | parental separation | 6yrs1m | 5 | 6 |
| Respondent's assessment of how much divorce since study child's 5th birthday has affected them | Parent | no | parental separation | 6yrs1m | 5 | 6 |
| Respondent's assessment of how much separating from partner since study child's 5th birthday has affected them | Parent | no | parental separation | 6yrs1m | 5 | 6 |
| Mother was divorced since the study child's 6th birthday | Parent | yes | parental separation | 9yrs2m | 6 | 7 |
| Mother and husband/partner separated since the study child's 6th birthday | Parent | yes | parental separation | 9yrs2m | 6 | 7 |
| Father was divorced since the study child's 6th birthday | Parent | yes | parental separation | 9yrs2m | 6 | 7 |
| Father and wife/partner separated since the study child's 6th birthday | Parent | yes | parental separation | 9yrs2m | 6 | 7 |
| Same partner/husband as mother had when study child had 6th birthday | Parent | no | parental separation | 9yrs2m | 6 | 9 |
| Same partner/wife as father had when study child had 6th birthday | Parent | no | parental separation | 9yrs2m | 6 | 9 |
| Mother was divorced since the study child's 6th birthday | Parent | no | parental separation | 9yrs2m | 8 | 9 |
| Mother and husband/partner separated since the study child's 6th birthday | Parent | no | parental separation | 9yrs2m | 8 | 9 |
| Father was divorced since the study child's 6th birthday | Parent | no | parental separation | 9yrs2m | 8 | 9 |
| Father and wife/partner separated since the study child's 6th birthday | Parent | no | parental separation | 9yrs2m | 8 | 9 |
| Respondent has divorced since the study child's 9th birthday | Parent | yes | parental separation | 11yrs2m | 9 | 10 |
| Respondent's wife/partner went away since the study child's 9th birthday | Parent | yes | parental separation | 11yrs2m | 9 | 10 |
| Respondent has separated from wife/partner since the study child's 9th birthday | Parent | yes | parental separation | 11yrs2m | 9 | 10 |
| Respondent has been divorced since child's 9th birthday | Parent | yes | parental separation | 11yrs2m | 9 | 10 |
| Respondent separated from husband/partner since the study child's 9th birthday | Parent | yes | parental separation | 11yrs2m | 9 | 10 |
| Partner's current partner is the same as on the study child's 9th birthday | Parent | no | parental separation | 12yrs1m | 9 | 12 |
| Mother's current partner is the same as on study child's 9th birthday | Parent | no | parental separation | 12yrs1m | 9 | 12 |
| Respondent has divorced since the study child's 9th birthday | Parent | no | parental separation | 11yrs2m | 11 | 11 |
| Respondent's wife/partner went away since the study child's 9th birthday | Parent | no | parental separation | 11yrs2m | 11 | 11 |
| Respondent has separated from wife/partner since the study child's 9th birthday | Parent | no | parental separation | 11yrs2m | 11 | 11 |
| Respondent has been divorced since child's 9th birthday | Parent | no | parental separation | 11yrs2m | 11 | 11 |
| Respondent separated from husband/partner since the study child's 9th birthday | Parent | no | parental separation | 11yrs2m | 11 | 11 |
| YP's parents have divorced/separated since the age of 12 | Child | no | parental separation | 16yrs | 12 | 16 |
| In the last year YP's parents have divorced | Child | no | parental separation | 17.5yrs | 16.5 | 17.5 |
| In the last year YP's parents have separated | Child | no | parental separation | 17.5yrs | 16.5 | 17.5 |
| Respondent was divorced in last year | Parent | no | parental separation | 2010 | 17.5 | 18.5 |
| Respondent and partner separated in last year | Parent | no | parental separation | 2010 | 17.5 | 18.5 |
| In last year, got divorced | Parent | no | parental separation | 2011-2013 | 19.5 | 20.5 |
| In last year, separated from partner | Parent | no | parental separation | 2011-2013 | 19.5 | 20.5 |
| PTNR physically cruel to CHDR >CH born | Parent | no | physical abuse | 8m | 0 | 0.67 |
| MUM physically cruel to CHDR >CH born | Parent | no | physical abuse | 8m | 0 | 0.67 |
| Frequency adult in family pushed, grabbed or shoved respondent before age of 11 | Child | yes | physical abuse | 22yrs | 0 | 11 |
| Frequency adult in family smacked respondent for discipline before age of 11 | Child | yes | physical abuse | 22yrs | 0 | 11 |
| Frequency adult in family actually kicked, punched, hit respondent with something that could hurt respondent or physically attacked respondent in another way before age of 11 | Child | yes | physical abuse | 22yrs | 0 | 11 |
| Frequency adult in family hit respondent so hard it left bruises or marks before age of 11 | Child | yes | physical abuse | 22yrs | 0 | 11 |
| When growing up people in respondent's family hit them so hard that it left them with bruises or marks | Child | yes | physical abuse | 23yrs | 0 | 16 |
| PTNR physical-cruel to CH since MID PREG | Parent | no | physical abuse | 8w | -1 | 0.16 |
| Ptnr Physically Cruel To Children | Parent | no | physical abuse | 8m | 0.67 | 0.67 |
| Self Physically Cruel To Children | Parent | no | physical abuse | 8m | 0.67 | 0.67 |
| Partner physically cruel to children >CH8MTHs | Parent | no | physical abuse | 1yrs9m | 0.67 | 2 |
| Mum physically cruel to children >CH8MTHs | Parent | no | physical abuse | 1yrs9m | 0.67 | 2 |
| Partner Physically Cruel to Child | Parent | no | physical abuse | 1yrs9m | 0.67 | 2 |
| Self Physically Cruel to Child | Parent | no | physical abuse | 1yrs9m | 0.67 | 2 |
| Whether partner was physically cruel to children since study child was 18 months old and effect this had | Parent | no | physical abuse | 2yrs9m | 1.5 | 3 |
| Whether mum was physically cruel to children since study child was 18 months old and effect this had | Parent | no | physical abuse | 2yrs9m | 1.5 | 3 |
| Partner's partner was physically cruel to their children since study child was 18 months old | Parent | no | physical abuse | 2yrs9m | 1.5 | 3 |
| Partner was physically cruel to their children since study child was 18 months old | Parent | no | physical abuse | 2yrs9m | 1.5 | 3 |
| PTR PHYS Cruel to CDRN> CH 30 MTHs | Parent | no | physical abuse | 3yrs11m | 2.5 | 4 |
| MUM PHYS Cruel to CDRN> CH 30 MTHs | Parent | no | physical abuse | 3yrs11m | 2.5 | 4 |
| Degree to which physical cruelty from a partner to children affected partner since study child was 2.5 years old | Parent | no | physical abuse | 3yrs11m | 2.5 | 4 |
| Degree to which partner being physically cruel to children affected partner since study child was 2.5 years old | Parent | no | physical abuse | 3yrs11m | 2.5 | 4 |
| Mothers partner was physically cruel to children in past year | Parent | no | physical abuse | 5yrs1m | 4 | 5 |
| Mother was physically cruel to children in past year | Parent | no | physical abuse | 5yrs1m | 4 | 5 |
| Respondent's assessment how much their partner being physically cruel to the children in the last year has affected them | Parent | no | physical abuse | 5yrs1m | 4 | 5 |
| Respondent's assessment of how much being physically cruel to the children in the last year has affected them | Parent | no | physical abuse | 5yrs1m | 4 | 5 |
| Respondent's partner physically cruel to respondent's children since study child's 5th birthday | Parent | no | physical abuse | 6yrs1m | 5 | 6 |
| Respondent physically cruel to own children since study child's 5th birthday | Parent | no | physical abuse | 6yrs1m | 5 | 6 |
| Respondent's assessment of how much partner's physical cruelty to children since study child's 5th birthday has affected them | Parent | no | physical abuse | 6yrs1m | 5 | 6 |
| Respondent's assessment of how much being physically cruel to children since study child's 5th birthday has affected them | Parent | no | physical abuse | 6yrs1m | 5 | 6 |
| Father's wife/partner was physically cruel to his children since the study child's 6th birthday | Parent | yes | physical abuse | 9yrs2m | 6 | 7 |
| Father was physically cruel to his children since the study child's 6th birthday | Parent | yes | physical abuse | 9yrs2m | 6 | 7 |
| Mother's husband/partner was physically cruel to her children since the study child's 6th birthday | Parent | yes | physical abuse | 9yrs2m | 6 | 7 |
| Mother was physically cruel to her children since the study child's 6th birthday | Parent | yes | physical abuse | 9yrs2m | 6 | 7 |
| Mother's husband/partner was physically cruel to her children since the study child's 6th birthday | Parent | no | physical abuse | 9yrs2m | 8 | 9 |
| Mother was physically cruel to her children since the study child's 6th birthday | Parent | no | physical abuse | 9yrs2m | 8 | 9 |
| Father's wife/partner was physically cruel to his children since the study child's 6th birthday | Parent | no | physical abuse | 9yrs2m | 8 | 9 |
| Father was physically cruel to his children since the study child's 6th birthday | Parent | no | physical abuse | 9yrs2m | 8 | 9 |
| Respondent's wife/partner was physically cruel to their children since the study child's 9th birthday | Parent | yes | physical abuse | 11yrs2m | 9 | 10 |
| Respondent was physically cruel to their children since the study child's 9th birthday | Parent | yes | physical abuse | 11yrs2m | 9 | 10 |
| Respondent's husband/partner was physically cruel to their children since study child's 9th birthday | Parent | yes | physical abuse | 11yrs2m | 9 | 10 |
| Respondent was physically cruel to their children since the study child's 9th birthday | Parent | yes | physical abuse | 11yrs2m | 9 | 10 |
| Respondent's wife/partner was physically cruel to their children since the study child's 9th birthday | Parent | no | physical abuse | 11yrs2m | 11 | 11 |
| Respondent was physically cruel to their children since the study child's 9th birthday | Parent | no | physical abuse | 11yrs2m | 11 | 11 |
| Respondent's husband/partner was physically cruel to their children since study child's 9th birthday | Parent | no | physical abuse | 11yrs2m | 11 | 11 |
| Respondent was physically cruel to their children since the study child's 9th birthday | Parent | no | physical abuse | 11yrs2m | 11 | 11 |
| Frequency adult in family pushed, grabbed or shoved respondent between ages of 11 and 17 | Child | yes | physical abuse | 22yrs | 11 | 17 |
| Frequency adult in family smacked respondent for discipline between ages of 11 and 17 | Child | yes | physical abuse | 22yrs | 11 | 17 |
| Frequency adult in family actually kicked, punched, hit respondent with something that could hurt respondent or physically attacked respondent in another way between ages of 11 and 17 | Child | yes | physical abuse | 22yrs | 11 | 17 |
| Frequency adult in family hit respondent so hard it left bruises or marks between ages of 11 and 17 | Child | yes | physical abuse | 22yrs | 11 | 17 |
| Respondent's partner was physically cruel to respondent's children in last year | Parent | no | physical abuse | 2010 | 17.5 | 18.5 |
| Respondent was physically cruel to own children in last year | Parent | no | physical abuse | 2010 | 17.5 | 18.5 |
| In last year, partner was physically cruel to respondent's children | Parent | no | physical abuse | 2011-2013 | 19.5 | 20.5 |
| In last year, respondent was physically cruel to respondent's children | Parent | no | physical abuse | 2011-2013 | 19.5 | 20.5 |
| Respondent was touched in a sexual way by adult or older child, or was forced to touch adult or older child in a sexual way, before age of 11 | Child | yes | sexual abuse | 22yrs | 0 | 11 |
| Adult or older child forced, or attempted to force, respondent into any sexual activity by threatening or holding respondent down or hurting respondent in some way, before age of 11 | Child | yes | sexual abuse | 22yrs | 0 | 11 |
| When growing up someone molested respondent (sexually) | Child | yes | sexual abuse | 23yrs | 0 | 16 |
| CH Sexually Abused > 6 MTHS | Parent | no | sexual abuse | 18m | 0.5 | 1.5 |
| Child sexually abused > 18 months, Y/N | Parent | no | sexual abuse | 30m | 1.5 | 2.5 |
| CH was Sexually Abused Past 12 MTHs | Parent | no | sexual abuse | 42m | 2.5 | 3.5 |
| Child was sexually abused since age 3 | Parent | no | sexual abuse | 57m | 3 | 5 |
| Child sexually abused in past 15 months | Parent | no | sexual abuse | 69m | 4.5 | 6 |
| Child was sexually abused since his/her 5th birthday | Parent | no | sexual abuse | 81m | 5 | 7 |
| Since 7th birthday child has been sexually abused | Parent | no | sexual abuse | 105m | 7 | 9 |
| Respondent was touched in a sexual way by adult or older child, or was forced to touch adult or older child in a sexual way, between ages of 11 and 17 | Child | yes | sexual abuse | 22yrs | 11 | 17 |
| Adult or older child forced, or attempted to force, respondent into any sexual activity by threatening or holding respondent down or hurting respondent in some way, between ages of 11 and 17 | Child | yes | sexual abuse | 22yrs | 11 | 17 |
| Smoked cannabis in 1-3MTHS of PREG | Parent | yes | substance household | 18w gest | -1 | -1 |
| Smoked cannabis >3MTHS PREG | Parent | no | substance household | 18w gest | -1 | -1 |
| Hard drugs | Parent | no | substance household | 18w gest | -1 | -1 |
| Had drug addiction | Parent | no | substance household | 15w gest | -1 | -1 |
| Had alcoholism | Parent | no | substance household | 15w gest | -1 | -1 |
| Had drug addiction | Parent | no | substance household | 12w gest | -1 | -1 |
| Had alcoholism | Parent | no | substance household | 12w gest | -1 | -1 |
| Hard drugs | Parent | no | substance household | 18w gest | -1 | -1 |
| FREQ of ganja use in last 2MTHS of PREG | Parent | yes | substance household | 8w | -1 | 0 |
| Hard drug in last 2 months | Parent | no | substance household | 8w | -1 | 0 |
| FREQ of ganja use since birth | Parent | no | substance household | 8w | 0 | 0.16 |
| Hard drug use since delivery | Parent | no | substance household | 8w | 0 | 0.16 |
| FREQ cannabis smoked since birth | Parent | no | substance household | 8w | 0 | 0.16 |
| Cannabis use since CH born | Parent | no | substance household | 8m | 0 | 0.67 |
| Amphetamine use since CH born | Parent | no | substance household | 8m | 0 | 0.67 |
| Opiate or cocaine use since CH born | Parent | no | substance household | 8m | 0 | 0.67 |
| PTNR had alcoholism >CH born | Parent | no | substance household | 8m | 0 | 0.67 |
| Used Cannabis/Marijuana Since Baby Born | Parent | no | substance household | 8m | 0 | 0.67 |
| Used Amphetamines Since Baby Born | Parent | no | substance household | 8m | 0 | 0.67 |
| Used Heroin, Cocaine Since Baby Born | Parent | no | substance household | 8m | 0 | 0.67 |
| Hard drugs | Parent | no | substance household | 8w | 0.16 | 0.16 |
| Mum had cannabis >CH8MTHs | Parent | no | substance household | 1yrs9m | 0.67 | 2 |
| Mum had amphetamines >CH8MTHs | Parent | no | substance household | 1yrs9m | 0.67 | 2 |
| Mum had heroin meth coc >CH8MTHs | Parent | no | substance household | 1yrs9m | 0.67 | 2 |
| Partner alcoholic >CH8MTHs | Parent | no | substance household | 1yrs9m | 0.67 | 2 |
| Taken Cannabis Since CH > 8 Months | Parent | no | substance household | 1yrs9m | 0.67 | 2 |
| Taken Amphetamines Since CH > 8 Months | Parent | no | substance household | 1yrs9m | 0.67 | 2 |
| Taken Heroin/Cocaine CH > 8 Months | Parent | no | substance household | 1yrs9m | 0.67 | 2 |
| Frequency Mum has taken cannabis since study child was 18 months old | Parent | no | substance household | 2yrs9m | 1.5 | 3 |
| Frequency Mum has taken amphetamines since study child was 18 months old | Parent | no | substance household | 2yrs9m | 1.5 | 3 |
| Frequency Mum has taken herion, methadone, crack or cocaine since study child was 18 months old | Parent | no | substance household | 2yrs9m | 1.5 | 3 |
| Partner had an alcohol problem since study child was 18 months old | Parent | no | substance household | 2yrs9m | 1.5 | 3 |
| Frequency partner has taken cannabis/marihuana since the study child was 18 months old | Parent | no | substance household | 2yrs9m | 1.5 | 3 |
| Frequency partner has taken amphetamines or other stimulants since the study child was 18 months old | Parent | no | substance household | 2yrs9m | 1.5 | 3 |
| Frequency partner has taken heroin/methadone/crack/cocaine since the study child was 18 months old | Parent | no | substance household | 2yrs9m | 1.5 | 3 |
| Frequency partner has used cannabis/marihuana in the past year | Parent | no | substance household | 3yrs11m | 3 | 4 |
| MUM took Cannabis >1 YR | Parent | no | substance household | 3yrs11m | 3 | 4 |
| MUM took Amphetemines >1 YR | Parent | no | substance household | 3yrs11m | 3 | 4 |
| MUM took Heroin >1 YR | Parent | no | substance household | 3yrs11m | 3 | 4 |
| Partner had Alcohol Problem> 1 YR | Parent | no | substance household | 3yrs11m | 3 | 4 |
| Frequency partner has taken amphetamines or other stimulants in the past year | Parent | no | substance household | 3yrs11m | 3 | 4 |
| Frequency partner has taken heroin, methadone, crack or cocaine in the past year | Parent | no | substance household | 3yrs11m | 3 | 4 |
| Mother had alcohol problem in past year | Parent | no | substance household | 5yrs1m | 4 | 5 |
| Frequency mother had cannabis/marijuana in past year | Parent | no | substance household | 5yrs1m | 4 | 5 |
| Frequency mother had amphetamines in past year | Parent | no | substance household | 5yrs1m | 4 | 5 |
| Frequency mother had heroin/methadone/cocaine in past year | Parent | no | substance household | 5yrs1m | 4 | 5 |
| Respondent had alcohol problems in the past year | Parent | no | substance household | 5yrs1m | 4 | 5 |
| Frequency in the past year respondent has taken cannabis/marihuana | Parent | no | substance household | 5yrs1m | 4 | 5 |
| Frequency in the past year respondent has taken amphetamines/other stimulants | Parent | no | substance household | 5yrs1m | 4 | 5 |
| Frequency in the past year respondent has taken heroin/methadone/crack/cocaine | Parent | no | substance household | 5yrs1m | 4 | 5 |
| Respondent has taken heroin, methadone, crack or cocaine since child's 5th birthday | Parent | no | substance household | 6yrs1m | 5 | 6 |
| Respondent has had/continued to have alcohol problem since study child's 5th birthday | Parent | no | substance household | 6yrs1m | 5 | 6 |
| Frequency respondent has taken cannabis/marihuana since study child's 5th birthday | Parent | no | substance household | 6yrs1m | 5 | 6 |
| Frequency respondent has taken amphetamines/other stimulants since study child's 5th birthday | Parent | no | substance household | 6yrs1m | 5 | 6 |
| Frequency respondent has taken heroin, methadone, crack, cocaine since study child's 5th birthday | Parent | no | substance household | 6yrs1m | 5 | 6 |
| Respondent's partner has had a drink (alcohol) problem since study child was 5 years old | Parent | no | substance household | 6yrs1m | 5 | 6 |
| Respondent has suffered from alcohol problems since child's 5th birthday | Parent | no | substance household | 6yrs1m | 5 | 6 |
| Respondent has taken cannabis or marihuana since child's 5th birthday | Parent | no | substance household | 6yrs1m | 5 | 6 |
| Respondent has taken amphetamines or other stimulants since child's 5th birthday | Parent | no | substance household | 6yrs1m | 5 | 6 |
| Respondent's partner has had alcohol problems since study child was 5 | Parent | no | substance household | 6yrs1m | 5 | 6 |
| Mother has had alcohol problem in last 3 years | Parent | no | substance household | 9yrs2m | 6 | 9 |
| Father has had alcohol problem in last 3 years | Parent | no | substance household | 9yrs2m | 6 | 9 |
| Frequency mother has taken cannabis/marihuana in last 2 years | Parent | no | substance household | 9yrs2m | 7 | 9 |
| Frequency mother has taken amphetamines or other stimulants in last 2 years | Parent | no | substance household | 9yrs2m | 7 | 9 |
| Frequency mother has taken heroin, methadone, crack, cocaine in last 2 years | Parent | no | substance household | 9yrs2m | 7 | 9 |
| Husband/partner has had drink (alcohol) problem, in last 2 years | Parent | no | substance household | 9yrs2m | 7 | 9 |
| Frequency father has taken cannabis/marihuana in last 2 years | Parent | no | substance household | 9yrs2m | 7 | 9 |
| Frequency father has taken amphetamines or other stimulants in last 2 years | Parent | no | substance household | 9yrs2m | 7 | 9 |
| Frequency father has taken heroin, methadone, crack, cocaine in last 2 years | Parent | no | substance household | 9yrs2m | 7 | 9 |
| Wife/partner has had drink (alcohol) problem, in last 2 years | Parent | no | substance household | 9yrs2m | 7 | 9 |
| Mother has ever had a drug addiction | Parent | no | substance household | 8yrs1m | 8 | 8 |
| Mother has ever had alcoholism | Parent | no | substance household | 8yrs1m | 8 | 8 |
| Respondent has ever had drug addiction | Parent | no | substance household | 8yrs1m | 8 | 8 |
| Respondent has ever had a drug addiction | Parent | no | substance household | 11yrs2m | 10 | 11 |
| Partner has had an alcohol problem in the last two years | Parent | no | substance household | 12yrs1m | 10 | 12 |
| Partner's partner had a drink problem in the last 2 years | Parent | no | substance household | 12yrs1m | 10 | 12 |
| Mother has had an alcohol problem in last 2 years | Parent | no | substance household | 12yrs1m | 10 | 12 |
| Mother's partner has had an alcohol problem since study child's 10th birthday | Parent | no | substance household | 12yrs1m | 10 | 12 |
| Respondent has ever had a drug addiction | Parent | no | substance household | 11yrs2m | 11 | 11 |
| Frequency respondent taken cannabis/marijuana in last two years | Parent | no | substance household | 2010 | 16.5 | 18.5 |
| Frequency respondent taken cocaine in last two years | Parent | no | substance household | 2010 | 16.5 | 18.5 |
| Frequency respondent taken amphetamines, ecstasy or other stimulants in last two years | Parent | no | substance household | 2010 | 16.5 | 18.5 |
| Frequency respondent taken heroin, methadone, crack or other hard drug in last two years | Parent | no | substance household | 2010 | 16.5 | 18.5 |
| Alcohol Use Disorders Identification Test (AUDIT) score | Child | no | substance household | 17.5yrs | 17.5 | 17.5 |
| AUDIT total score | Parent | no | substance household | 2010 | 18.5 | 18.5 |
| Cannabis/marijuana | Parent | no | substance household | 2011-2013 | 18.5 | 20.5 |
| Cocaine | Parent | no | substance household | 2011-2013 | 18.5 | 20.5 |
| Amphetamines, ecstasy or other stimulants | Parent | no | substance household | 2011-2013 | 18.5 | 20.5 |
| Heroin, methadone, crack, other hard drug | Parent | no | substance household | 2011-2013 | 18.5 | 20.5 |
| AUDIT total score | Parent | no | substance household | 2011-2013 | 20.5 | 20.5 |
| Physically hurt by PTNR >CH born | Parent | no | violence between parents | 8m | 0 | 0.67 |
| Ptnr Physically Cruel Since Baby Born | Parent | no | violence between parents | 8m | 0 | 0.67 |
| Amount YP has ever been aware of and affected by one 'parent' slapping, kicking, hitting or otherwise physically hurting the other | Child | yes | violence between parents | 21yrs | 0 | 21 |
| PTNR physically hurt you since MID PREG | Parent | no | violence between parents | 8w | -1 | 0.16 |
| Partner physically cruel to mum >CH8MTHs | Parent | no | violence between parents | 1yrs9m | 0.67 | 2 |
| Partner Physically Cruel | Parent | no | violence between parents | 1yrs9m | 0.67 | 2 |
| Whether partner was physically cruel to mum since study child was 18 months old and effect this had | Parent | no | violence between parents | 2yrs9m | 1.5 | 3 |
| Partner's partner was physically cruel to them since study child was 18 months old | Parent | no | violence between parents | 2yrs9m | 1.5 | 3 |
| PTR PHYS Cruel to MUM> CH 30 MTHs | Parent | no | violence between parents | 3yrs11m | 2.5 | 4 |
| Degree to which physical cruelty from a partner affected partner since child was 2.5 years old | Parent | no | violence between parents | 3yrs11m | 2.5 | 4 |
| Mothers partner was physically cruel to her in past year | Parent | no | violence between parents | 5yrs1m | 4 | 5 |
| Respondent's assessment of how much their partner being physically cruel in the last year has affected them | Parent | no | violence between parents | 5yrs1m | 4 | 5 |
| Respondent's partner was physically cruel to them since study child's 5th birthday | Parent | no | violence between parents | 6yrs1m | 5 | 6 |
| Respondent's assessment of how much partner being physically cruel since study child's 5th birthday has affected them | Parent | no | violence between parents | 6yrs1m | 5 | 6 |
| Mother's husband/partner was physically cruel to her since the study child's 6th birthday | Parent | yes | violence between parents | 9yrs2m | 6 | 7 |
| Father's wife/partner was physically cruel to him since the study child's 6th birthday | Parent | yes | violence between parents | 9yrs2m | 6 | 7 |
| Mother has ever kicked, bitten or hit partner with a fist | Parent | no | violence between parents | 8yrs1m | 8 | 8 |
| Partner has ever kicked, bitten or hit mother with a fist | Parent | no | violence between parents | 8yrs1m | 8 | 8 |
| Mother has ever physically twisted partner's arm | Parent | no | violence between parents | 8yrs1m | 8 | 8 |
| Partner has ever physically twisted mother's arm | Parent | no | violence between parents | 8yrs1m | 8 | 8 |
| Mother has ever tried to throw partner bodily | Parent | no | violence between parents | 8yrs1m | 8 | 8 |
| Partner has ever tried to throw mother bodily | Parent | no | violence between parents | 8yrs1m | 8 | 8 |
| Mother has ever beaten partner up | Parent | no | violence between parents | 8yrs1m | 8 | 8 |
| Partner has ever beaten mother up | Parent | no | violence between parents | 8yrs1m | 8 | 8 |
| Mother has ever tried to choke or strangle partner | Parent | no | violence between parents | 8yrs1m | 8 | 8 |
| Partner has ever tried to choke or strangle mother | Parent | no | violence between parents | 8yrs1m | 8 | 8 |
| Mother has ever threatened partner with a knife or other weapon | Parent | no | violence between parents | 8yrs1m | 8 | 8 |
| Partner has ever threatened mother with a knife or other weapon | Parent | no | violence between parents | 8yrs1m | 8 | 8 |
| Mother has ever used a knife or other weapon on partner | Parent | no | violence between parents | 8yrs1m | 8 | 8 |
| Partner has ever used a knife or other weapon on mother | Parent | no | violence between parents | 8yrs1m | 8 | 8 |
| Respondent has ever bitten/kicked/hit their partner with a fist | Parent | no | violence between parents | 8yrs1m | 8 | 8 |
| Respondent's partner has ever bitten/kicked/hit them with a fist | Parent | no | violence between parents | 8yrs1m | 8 | 8 |
| Respondent has tried to twist their partner's arm | Parent | no | violence between parents | 8yrs1m | 8 | 8 |
| Respondent's partner has tried to twist their arm | Parent | no | violence between parents | 8yrs1m | 8 | 8 |
| Respondent has ever thrown/tried to throw their partner | Parent | no | violence between parents | 8yrs1m | 8 | 8 |
| Respondent's partner has ever thrown/tried to throw them | Parent | no | violence between parents | 8yrs1m | 8 | 8 |
| Respondent has ever beaten up their partner | Parent | no | violence between parents | 8yrs1m | 8 | 8 |
| Respondent's partner has ever beaten them up | Parent | no | violence between parents | 8yrs1m | 8 | 8 |
| Respondent has ever tried to choke their partner | Parent | no | violence between parents | 8yrs1m | 8 | 8 |
| Respondent's partner has ever tried to choke them | Parent | no | violence between parents | 8yrs1m | 8 | 8 |
| Respondent has ever threatened their partner with a knife/weapon | Parent | no | violence between parents | 8yrs1m | 8 | 8 |
| Respondent's partner has ever threatened them with a knife/weapon | Parent | no | violence between parents | 8yrs1m | 8 | 8 |
| Respondent has ever used a knife/weapon on their partner | Parent | no | violence between parents | 8yrs1m | 8 | 8 |
| Respondent's partner has ever used a knife/weapon on them | Parent | no | violence between parents | 8yrs1m | 8 | 8 |
| Mother's husband/partner was physically cruel to her since the study child's 6th birthday | Parent | no | violence between parents | 9yrs2m | 8 | 9 |
| Father's wife/partner was physically cruel to him since the study child's 6th birthday | Parent | no | violence between parents | 9yrs2m | 8 | 9 |
| Respondent's wife/partner was physically cruel to them since the study child's 9th birthday | Parent | yes | violence between parents | 11yrs2m | 9 | 10 |
| Respondent's husband/partner was physically cruel to them since study child's 9th birthday | Parent | yes | violence between parents | 11yrs2m | 9 | 10 |
| Respondent's wife/partner was physically cruel to them since the study child's 9th birthday | Parent | no | violence between parents | 11yrs2m | 11 | 11 |
| Respondent's husband/partner was physically cruel to them since study child's 9th birthday | Parent | no | violence between parents | 11yrs2m | 11 | 11 |
| Respondent's partner was physically cruel to respondent in last year | Parent | no | violence between parents | 2010 | 17.5 | 18.5 |
| In last year, partner was physically cruel to respondent | Parent | no | violence between parents | 2011-2013 | 20.5 | 20.5 |

| **Table S3.** Multiple Health Risk Behaviours (MRBs). | |
| --- | --- |
| **MRBs** | **Definition/how derived** |
| Physical inactivity | Young person (YP) has typically over the past year exercised <5times per week. The UK guidance for physical activity [1] in children and YP is that moderate to vigorous intensity physical activity for at least 60 minutes and up to several hours every day should be undertaken. |
| Excessive TV viewing | YP spent 3 or more hours watching TV on average per day across the week. |
| Car passenger risk | YP had been in a car passenger at least once in their lifetime where the driver (1) had consumed alcohol or (2) did not have a valid licence, or (3) the YP chose not to wear a seat belt last time travelled in a car, van or taxi. |
| Cycle helmet use | If the YP reported that they had last ridden a bicycle within the previous 4 weeks and they had not worn a helmet on the most recent occasion. |
| Scooter risk | YP has driven a motorbike/scooter off road, or without a licence on a public road at least once. |
| Criminal/antisocial behaviour | YP reported that at least once in the past year they had undertaken at least one of the following seven offences: carried a weapon; physically hurt someone on purpose; stolen something; sold illicit substances to another person; damaged property belonging to someone else either by using graffiti, setting fire to it or destroying or damaging it in another fashion; subjected someone to verbal or physical racial abuse; or been rude/rowdy in a public place. |
| Hazardous alcohol consumption | In the past year had scored 8 or more on the Alcohol Use Disorders Identification Test indicating hazardous alcohol consumption. |
| Regular tobacco smoking | Has ever smoked and is regularly smoking by currently smoking at least one cigarette per week. |
| Cannabis use | Those who reported using cannabis ‘sometimes but less often than once a week’ or more regular use were classified as occasional users. |
| Illicit drug/solvent use | In the year since their 15th birthday, YP had either been a regular user (ie, used five or more times) of one or more illicit drugs (excluding cannabis) including amphetamines, ecstasy, lysergic acid diethylamide (LSD), cocaine, ketamine or inhalants including aerosols, gas, solvents and poppers. |
| Self-harm | Young people who said they had purposely hurt themselves in some way in their lifetime. |
| Penetrative sex before age 16 | YP reported having had penetrative sex in the preceding year and that they were under 16 at the time. |
| Unprotected sex | Penetrative sex without the use of contraception on the last occasion they had had sex in the past year. |

## Sources of information:

## Age 15 years clinic: criminal and antisocial behaviour, penetrative sex prior to age 16, and unprotected sex.

## Age 16 years questionnaire: physical inactivity, excessive TV viewing, car passenger risk, cycle helmet use, scooter risk, hazardous alcohol drinking, regular smoking, illicit drug/solvent use and self-harm.

[1] Department of Health. UK physical activity guidelines. London, 2011

## **Table S4.** Questions that populate subscales on the parent-report version of the Strengths and Difficulties Questionnaire for 4-17 year olds.

| Emotional problems scale | Often complains of headaches, stomach-aches or sicknessMany worries, often seems worriedOften unhappy, downhearted or tearfulNervous or clingy in new situations, easily loses confidenceMany fears, easily scared |
| --- | --- |
| Conduct problems Scale | Often has temper tantrums or hot tempersGenerally obedient, usually does what adults requestOften fights with other children or bullies themOften lies or cheatsSteals from home, school or elsewhere |
| Hyperactivity scale | Restless, overactive, cannot stay still for longConstantly fidgeting or squirmingEasily distracted, concentration wandersThinks things out before actingSees tasks through to the end, good attention span |
| Peer problems scale | Rather solitary, tends to play aloneHas at least one good friendGenerally liked by other childrenPicked on or bullied by other childrenGets on better with adults than with other children |
| Prosocial scale | Considerate of other people's feelingsShares readily with other children (treats, toys, pencils etc.)Helpful if someone is hurt, upset or feeling illKind to younger childrenOften volunteers to help others (parents, teachers, other children) |

## **Table S5.** Frequencies of adversities in complete case study sample (*N*=1348).

| **Adversities** | ***N*** | **Percent** |
| --- | --- | --- |
| Parent mental health problems or suicide | 423 | 31.38 |
| Violence between parents | 200 | 14.84 |
| Emotional abuse | 194 | 14.39 |
| Parental separation | 158 | 11.72 |
| Child experiences bullying | 132 | 9.79 |
| Physical abuse | 93 | 6.90 |
| Parent substance use | 89 | 6.6 |
| Parent criminal conviction | 77 | 5.71 |
| Sexual abuse | 5 | 0.37 |

Note: For definitions of each adversity see Table S1 above.

## **Table S6.** Tetrachoric correlations between 9 adversities (unimputed data, *N*=3965).

|  | Sexual abuse | Physical abuse | Emotional abuse | Parent substance use | Parent mental health problems or suicide | Violence between parents | Parental separation | Child experiences bullying |
| --- | --- | --- | --- | --- | --- | --- | --- | --- |
|  |  |  |  |  |  |  |  |  |
| Sexual abuse |  |  |  |  |  |  |  |  |
| Physical abuse | 0.27 |  |  |  |  |  |  |  |
| Emotional abuse | 0.16 | 0.72 |  |  |  |  |  |  |
| Parent substance use | 0.07 | 0.14 | 0.25 |  |  |  |  |  |
| Parent mental health problems or suicide | -0.06 | 0.31 | 0.38 | 0.20 |  |  |  |  |
| Violence between parents | 0.13 | 0.42 | 0.43 | 0.23 | 0.31 |  |  |  |
| Parental separation | 0.34 | 0.23 | 0.34 | 0.21 | 0.26 | 0.36 |  |  |
| Child experiences bullying | 0.02 | 0.07 | 0.06 | 0.01 | 0.07 | 0.13 | 0.07 |  |
| Parent criminal conviction | 0.12 | 0.28 | 0.14 | 0.17 | 0.12 | 0.20 | 0.13 | 0.13 |

Note: For definitions of each adversity see Table S1.

**Table S7.** Tetrachoric correlations between 13 risk behaviours (unimputed data, *N*=2656).

|  | Physical inactivity | Excessive TV viewing | Passenger risk | Helmet use | Scooter risk | Criminal/ASB | Alcohol | Tobacco | Cannabis | Drugs | Self-harm | Sex prior to age 16 |
| --- | --- | --- | --- | --- | --- | --- | --- | --- | --- | --- | --- | --- |
| Physical inactivity |  |  |  |  |  |  |  |  |  |  |  |  |
| Excessive TV viewing | 0.03 |  |  |  |  |  |  |  |  |  |  |  |
| Passenger risk | 0.04 | 0.06 |  |  |  |  |  |  |  |  |  |  |
| Helmet use | -0.20 | -0.07 | 0.00 |  |  |  |  |  |  |  |  |  |
| Scooter risk | -0.13 | -0.02 | 0.24 | 0.32 |  |  |  |  |  |  |  |  |
| Criminal/ASB | -0.04 | 0.07 | 0.33 | 0.12 | 0.34 |  |  |  |  |  |  |  |
| Alcohol | 0.03 | 0.04 | 0.42 | 0.04 | 0.22 | 0.45 |  |  |  |  |  |  |
| Tobacco | 0.20 | 0.07 | 0.41 | 0.01 | 0.28 | 0.52 | 0.59 |  |  |  |  |  |
| Cannabis | 0.08 | -0.04 | 0.40 | 0.19 | 0.23 | 0.50 | 0.56 | 0.71 |  |  |  |  |
| Drugs | 0.08 | 0.01 | 0.35 | 0.16 | 0.17 | 0.44 | 0.49 | 0.58 | 0.74 |  |  |  |
| Self-harm | 0.17 | -0.03 | 0.27 | -0.11 | -0.04 | 0.33 | 0.32 | 0.41 | 0.35 | 0.46 |  |  |
| Sex prior to age 16 | 0.03 | 0.05 | 0.27 | -0.04 | 0.19 | 0.43 | 0.34 | 0.44 | 0.32 | 0.30 | 0.28 |  |
| Unprotected sex | 0.07 | 0.01 | 0.19 | -0.01 | 0.00 | 0.42 | 0.29 | 0.21 | 0.34 | 0.25 | 0.44 | 1.00 |

Note: For definitions of each risk behaviour see Table S4. ASB = antisocial behaviour.

## **Table S8.** Tetrachoric correlations between 9 adversities and 13 risk behaviours (unimputed data, *N*=1824).

|  | Physical abuse | Sexual abuse | Emotional abuse | Child experiences bullying | Violence between parents | Parent substance use | Parent mental health problems or suicide | Parent criminal conviction | Parental separation |
| --- | --- | --- | --- | --- | --- | --- | --- | --- | --- |
| Physical inactivity | -0.06 | 0.17 | -0.01 | -0.01 | -0.08 | 0.03 | 0.05 | 0.01 | 0.01 |
| Excessive TV viewing | -0.06 | 0.21 | 0.09 | -0.06 | 0.00 | 0.14 | 0.07 | -0.03 | 0.16 |
| Passenger risk | -0.02 | -0.01 | 0.15 | 0.09 | 0.05 | 0.20 | 0.03 | 0.10 | 0.15 |
| Helmet use | 0.09 | 0.01 | 0.01 | 0.00 | 0.07 | 0.12 | 0.00 | 0.07 | -0.04 |
| Scooter risk | -0.04 | -1.00 | 0.04 | 0.12 | 0.07 | 0.07 | -0.04 | 0.10 | 0.23 |
| Criminal/ASB | 0.13 | 0.08 | 0.12 | 0.11 | 0.10 | 0.05 | 0.07 | 0.03 | 0.09 |
| Alcohol | 0.03 | -0.24 | 0.04 | 0.02 | 0.12 | 0.15 | 0.04 | 0.10 | 0.07 |
| Tobacco | -0.10 | 0.22 | 0.04 | 0.09 | 0.00 | 0.13 | 0.06 | 0.08 | 0.22 |
| Cannabis | 0.00 | -1.00 | 0.11 | 0.02 | 0.02 | 0.14 | 0.08 | 0.07 | 0.09 |
| Drugs | 0.10 | -1.00 | 0.07 | 0.21 | 0.07 | 0.22 | 0.03 | -0.10 | 0.13 |
| Self-harm | 0.05 | 0.20 | 0.18 | 0.17 | 0.13 | 0.07 | 0.08 | 0.02 | 0.09 |
| Sex prior to age 16 | 0.13 | 0.16 | 0.08 | 0.10 | 0.14 | 0.00 | 0.09 | 0.12 | 0.12 |
| Unprotected sex | 0.04 | -1.00 | 0.14 | 0.18 | 0.06 | -0.11 | 0.16 | 0.07 | 0.13 |

Note: For definitions of each adversity see Table S1 and risk behaviour see Table S4. ASB = antisocial behaviour.

**Table S9.** Associations between each risk behaviour and total adversities (imputed data, *N*=5799).

|  | **Associations with total adversities (adjusted)** | | |
| --- | --- | --- | --- |
| **MRBs** | β | 95% CI | *p* |
| Physical inactivity | -0.03 | -0.14, 0.09 | 0.651 |
| Excessive TV viewing | 0.18 | 0.05, 0.30 | 0.007 |
| Passenger risk | 0.44 | 0.33, 0.55 | <0.001 |
| Helmet use | 0.14 | 0.03, 0.26 | 0.016 |
| Scooter risk | 0.34 | 0.20, 0.47 | 0.071 |
| Criminal/ASB | 0.36 | 0.26, 0.45 | <0.001 |
| Alcohol | 0.26 | 0.16, 0.37 | <0.001 |
| Tobacco | 0.61 | 0.46, 0.77 | <0.001 |
| Cannabis | 0.52 | 0.34, 0.70 | <0.001 |
| Drugs | 0.62 | 0.36, 0.89 | <0.001 |
| Self-harm | 0.47 | 0.33, 0.60 | <0.001 |
| Sex prior to age 16 | 0.32 | 0.18, 0.47 | <0.001 |
| Unprotected sex | 0.36 | -0.08, 0.79 | 0.110 |

Note: For definitions of each risk behaviour, see Table S4. 750 imputed data sets. CI, confidence interval, β, beta. Associations between each risk behaviour and total adversities modelled in linear regressions controlling for child sex, housing tenure, income, social class, maternal education, maternal age at birth and child IQ. Adjusted significance level due to multiple testing p < 0.0038.

**Table S10.** Missingness of adversities and risk behaviours (unimputed data, *N*=5799).

| **Adversities** | **% missing** | **MRBs** | **% missing** |
| --- | --- | --- | --- |
| Child experiences bullying | 24.33 | Scooter risk | 42.68 |
| Violence between parents | 23.57 | Drugs | 42.59 |
| Emotional abuse | 22.69 | Alcohol | 42.02 |
| Parental separation | 16.97 | Excessive tv viewing | 41.97 |
| Parent substance use | 15.55 | Sex prior to age 16 | 40.89 |
| Parent mental health problems or suicide | 14.26 | Unprotected sex | 40.56 |
| Physical abuse | 13.19 | Physical inactivity | 40.16 |
| Parent criminal conviction | 11.97 | Passenger risk | 39.85 |
| Sexual abuse | 5.74 | Helmet use | 39.85 |
|  |  | Cannabis | 39.82 |
|  |  | Tobacco | 39.77 |
|  |  | Self-harm | 39.70 |
|  |  | Criminal/ASB | 38.39 |

Note: For definitions of each adversity see Table S1 and risk behaviour see Table S4. ASB = antisocial behaviour.

Note: 55.6% of participants had complete ACE data, 29% had 1-3 missing values, 4% had 4-6 missing values and 11.4% had 7-9 missing values. 36% of participants had complete MRB data, 21.3% had 1-3 missing values, 3.3% had 4-6 missing values, 14.3% had 7-9 missing values and 25.1% had 10-13 missing values. Within this sample, each risk behaviour suffered from ∼40% missing data.

**Table S11.** Associations between adversities, sex, an interaction between sex and adversities and risk behaviours.

|  | **MRBs (adjusted)** | | | |
| --- | --- | --- | --- | --- |
|  | **Imputed sample (*N*=5799)** | | **Complete case sample (*N*=1348)** | |
|  | *ß* (95% CI) | *p-value* | *ß* (95% CI) | *p-value* |
| Adversities | 0.30 (0.21, 0.38) | <0.001 | 0.15 (0.02, 0.28) | 0.028 |
| Sex (male) | -0.13 (-0.31, 0.05) | 0.158 | -0.08 (-0.35, 0.31) | 0.562 |
| Sex (male)*adversities | 0.02 (-0.09, 0.13) | 0.738 | 0.01 (-0.08, 0.27) | 0.275 |

Note: Results adjusted for housing tenure, income, social class, maternal education, maternal age at birth and child IQ.
